# Influences on NHS Health Check behaviours: a systematic review

**DOI:** 10.1186/s12889-020-09365-2

**Published:** 2020-09-17

**Authors:** Lou Atkins, Chryssa Stefanidou, Tim Chadborn, Katherine Thompson, Susan Michie, Fabi Lorencatto

**Affiliations:** 1 Centre for Behaviour Change, University College London, WC1N 3AZ, London, UK; 2 Public Health England Behavioural Insights, London, UK

**Keywords:** NHS health check, Cardiovascular disease, Behaviour change wheel, Behaviour change techniques, Theoretical domains framework

## Abstract

**Background:**

National Health Service Health Checks were introduced in 2009 to reduce cardiovascular disease (CVD) risks and events. Since then, national evaluations have highlighted the need to maximise the programme’s impact by improving coverage and outputs. To address these challenges it is important to understand the extent to which positive behaviours are influenced across the NHS Health Check pathway and encourage the promotion or minimisation of behavioural facilitators and barriers respectively. This study applied behavioural science frameworks to: i) identify behaviours and actors relevant to uptake, delivery and follow up of NHS Health Checks and influences on these behaviours and; ii) signpost to example intervention content.

**Methods:**

A systematic review of studies reporting behaviours related to NHS Health Check-related behaviours of patients, health care professionals (HCPs) and commissioners. Influences on behaviours were coded using theory-based models: COM-B and Theoretical Domains Framework (TDF). Potential intervention types and behaviour change techniques (BCTs) were suggested to target key influences.

**Results:**

We identified 37 studies reporting nine behaviours and influences for eight of these. The most frequently identified influences were physical opportunity including HCPs having space and time to deliver NHS Health Checks and patients having money to adhere to recommendations to change diet and physical activity. Other key influences were motivational, such as beliefs about consequences about the value of NHS Health Checks and behaviour change, and social, such as influences of others on behaviour change. The following techniques are suggested for websites or smartphone apps: *Adding objects to the environment,* e.g. provide HCPs with electronic schedules to guide timely delivery of Health Checks to target physical opportunity, *Social support (unspecified)*, e.g. include text suggesting patients to ask a colleague to agree in advance to join them in taking the ‘healthy option’ lunch at work; *Information about health consequences*, e.g. quotes and/or videos from patients talking about the health benefits of changes they have made.

**Conclusions:**

Through the application of behavioural science we identified key behaviours and their influences which informed recommendations for intervention content. To ascertain the extent to which this reflects existing interventions we recommend a review of relevant evidence.

## Background

In England in 2017, more than 124,000 people died from cardiovascular disease (CVD).^1^ Changing behaviours related to diet, physical activity, smoking and alcohol intake can reduce CVD risk. The delivery of interventions targeting these behaviours also often requires healthcare professional (HCP) behaviours to change.

In 2009, the English National Health Service (NHS) launched ‘NHS Health Check’, a national prevention programme offered to adults 40–74 years old with the aim of helping them reduce their chance of having a heart attack or stroke through behaviour change and, where appropriate, clinical treatment. In brief, eligible patients attending an NHS Health Check will have seven risk factors measured and their 10-year risk of CVD calculated as part of an appointment lasting around 20 min (the majority of which are delivered by healthcare assistants in primary care). During the appointment these results are discussed and the individual is supported to make behaviour changes and/or access clinical treatment to reduce their risk of stroke, kidney disease, heart disease, diabetes or dementia. The programme standards have been developed to guide implementation and delivery of NHS Health Checks [1]. Cost-effectiveness calculations for this programme were based on an assumed uptake of 75% of all those eligible [2]. However, since 2013 when delivery of NHS Health Check became a statutory responsibility of local authorities, < 50% of those eligible have received a NHS Health Check [3]. Improving the effectiveness and uptake of the programme is a key part of PHE’s strategic priority around predictive prevention to better predict and prevent poor health. Interventions are more likely to be effective if they target influences on behaviour [4]. So it needs to be established what the behaviours relevant to NHS Health Checks are, who performs them and the factors influencing these behaviours. To date, research has tended to focused on single populations, e.g. patients or GPs, and specific behaviours, e.g. attending an NHS Health Check. A synthesis of these studies would provide an overarching behavioural picture of those involved in delivery and receipt of NHS Health Checks and so provide the foundations for intervention refinement and development.

**Fig. 1:**
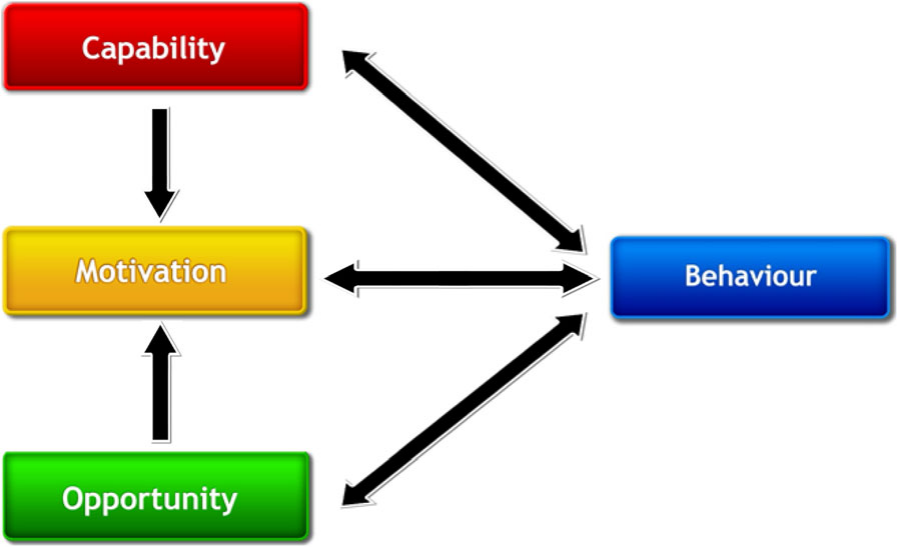
COM-B model

**Fig. 2:**
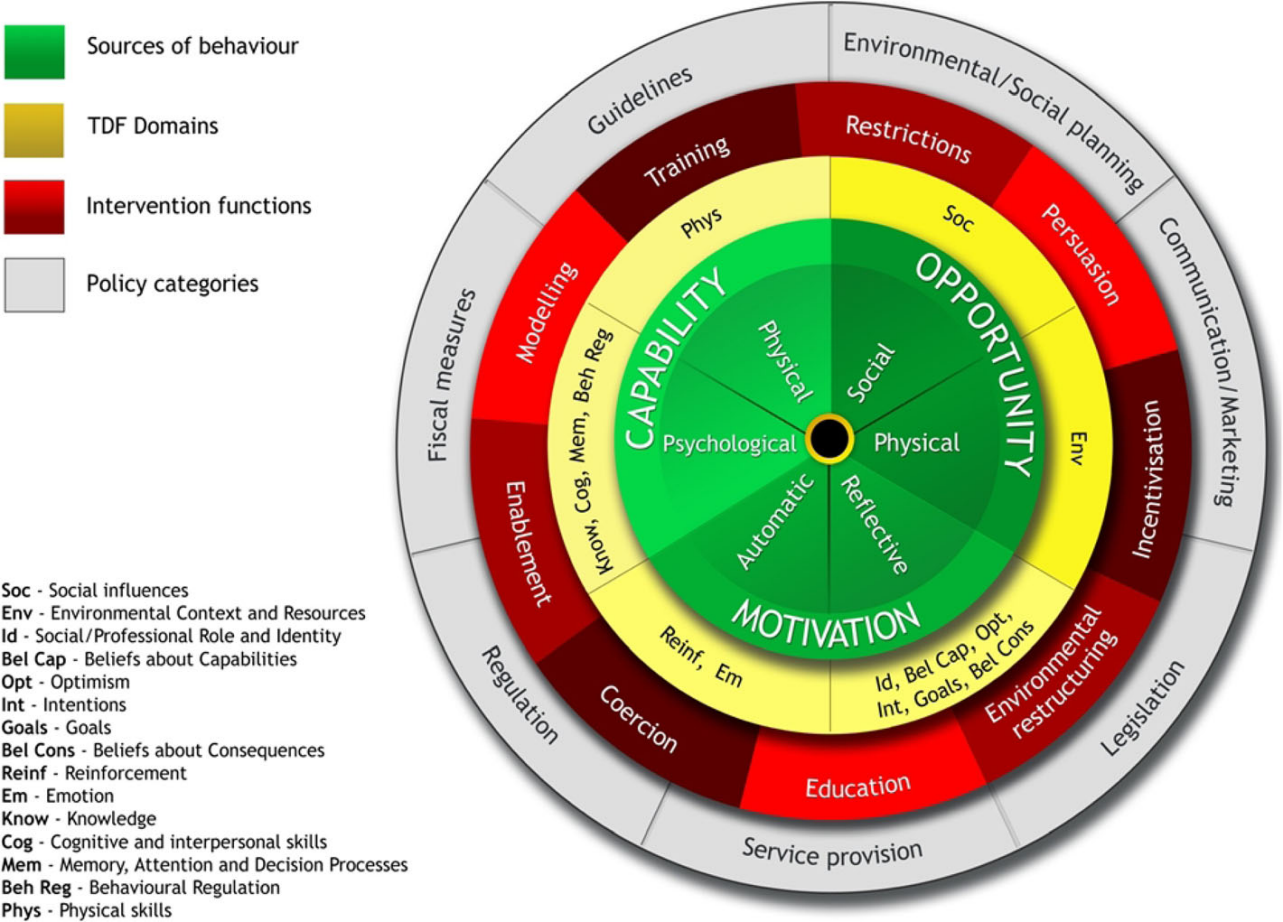
TDF domains linked to COM-B within the Behaviour Change Wheel

Tools such as the Behaviour Change Wheel (BCW) [5], which includes the theoretical model of behaviour COM-B (Fig. 1); the Theoretical Domains Framework (TDF) (Fig. 2 shows how the TDF domains are linked to each COM-B component (see Additional file 1 for labels and definitions)) [4, 6] and the Behaviour Change Techniques Taxonomy (BCTTv1) [7] can be used for identifying influences on behaviours (COM-B and TDF) and providing recommendations for intervention design based on the influences identified (BCW and BCTTv1). The COM-B model, which sits at the ‘hub’ of the BCW, is a simple model to understand behaviour in terms of the Capability, Opportunity and Motivation needed to perform a Behaviour (Fig. 1).

The TDF is used as a framework for synthesising behavioural influences in systematic literature reviews across qualitative and quantitative studies reporting perceived barriers and facilitators of behaviours. These include increasing attendance to diabetic retinopathy screening and triage [8], treatment and transfer of acute stroke patients in emergency care settings [9] and uptake of weight-management programmes in adults at risk of type 2 diabetes [10].

The Behaviour Change Wheel (BCW), a synthesis of 19 frameworks of behaviour change, can be used to characterise interventions. COM-B sits at the ‘hub’ of the Wheel and is surrounded by nine broad types of intervention and seven policy options, i.e. channels through which interventions are implemented (Fig. 2; see Additional file 2 for labels and definitions and Additional file 3 for links between influences on behaviour and potential intervention content.

How intervention functions are delivered can be described using a 93-item taxonomy of behaviour change techniques (BCTTv1) [6]. Behaviour change techniques (BCTs) are defined as the active ingredients in interventions designed to bring about change. The Theory and Techniques Tool (https://theoryandtechniquetool.humanbehaviourchange.org/ - see Additional file 4) articulates the strength of evidence between BCTs and their hypothesised mechanisms of action.

The aims of this study were to identify:

1. groups of people (actors) and their behaviours that are relevant to increasing uptake and follow up of NHS Health Checks within primary care and community and/or social care, representing these in a conceptual, ‘systems’ map.
2. influences on the behaviours identified and categorise them using two theoretical models: COM-B and TDF.
3. types of intervention and component behaviour change techniques (BCTs) likely to change these influences.

## Methods

### Search strategy and selection criteria

We conducted a systematic review in accordance with PRISMA guidelines. Electronic databases Medline, EMBASE and PsycINFO were searched for publications reporting barriers to and facilitators of behaviours relevant to uptake and follow up of the NHS Health Check. We included empirical qualitative and/or quantitative research and systematic review articles of behaviours and of barriers to and facilitators of behaviours relevant to NHS Health Checks. We included all papers with title and abstract written in English. Searches were limited to 2008 - December 2018 as this corresponded to the introduction of NHS Health Checks. The full search strategy is provided in Additional file 5.

### Stakeholder consultation

We assembled a panel of stakeholder experts to suggest relevant literature not identified by the electronic searches.

### Study selection and quality assessment

Titles and abstracts were screened against the inclusion and exclusion criteria by two researchers. For selected abstracts and those where there was uncertainty at first screening, full papers were screened against the same inclusion and exclusion criteria. Any papers for which the decision was not clear were discussed with other members of the review group. We used the Mixed Methods Appraisal Tool (MMAT) [11] to assess quality of qualitative, quantitative and mixed methods studies (Additional file 6).

### Data extraction tools

Study characteristics extracted were: setting; participant; target behaviour; how the target behaviour was measured and barriers and facilitators to the target behaviour. Quotes and author interpretations of barriers and facilitators were coded using COM-B and TDF.

### Data analysis

We conducted a six-step framework [12] and thematic [13] analysis to synthesise and explain influences on NHS Health Check related behaviours identified in the systematic review:

i) Framework analysis by deductively coding extracted data on barriers/facilitators into the COM-B and TDF domain(s) they were judged to best represent.
ii) Thematic analysis within each domain, grouping similar data points and inductively generating summary theme labels.
iii) Recording the frequency of each theme, i.e. how many studies each theme was identified in.
iv) Classifying each theme as either barrier, facilitator, or both.
v) Classifying each domain as either barrier (all identified themes within that domain are barriers), facilitator (all identified themes within that domain are facilitators) or both (if themes within the domain are a combination of barriers and facilitators).
vi) Selecting key themes within domains using: i) established criteria - frequency (number of studies), elaboration (number of themes) and evidence of conflicting beliefs within domains (e.g. if some participants report lack of knowledge of guidelines whereas others report familiarity with guidelines) [14]; ii) PHE stakeholder input to identify strategic priority areas to target.

TDF provides a greater level of detail than COM-B and was used primarily to classify influences on behaviours.

Using the Behaviour Change Wheel, based on COM-B and TDF coded influences on behaviours, we signposted to functions interventions might serve [15] and BCTs to deliver those functions [16]. The following steps were taken to translate the list of potentially relevant BCTs into the recommendations for intervention design and refinement:

i) Stakeholders with relevant perspectives on the delivery of these BCTs selected BCTs for a prototype intervention.To permit a systematic approach to selecting which BCTs are appropriate to each context, the APEASE criteria [13] were used. These form a checklist of considerations when selecting intervention content and mode of delivery, i.e. is it Affordable - can it be delivered to budget?; Practicable - can it be delivered to scale?; Effective/Cost-effective - is there evidence it is likely to be (cost)effective?; Acceptable - is it acceptable to those delivering, receiving and commissioning it?; are there any Side-effects/Safety issues?; will it increase Equity, i.e. does it disadvantage any groups? See Additional file 7.
ii) Having produced a prototype intervention, further feedback (again guided by the APEASE criteria) was obtained from a wider group of stakeholders including HCPs (GPs, practice nurses, practice managers and commissioners) and patients (NHS Health Check attenders and non-attenders).

## Results

**Fig. 3:**
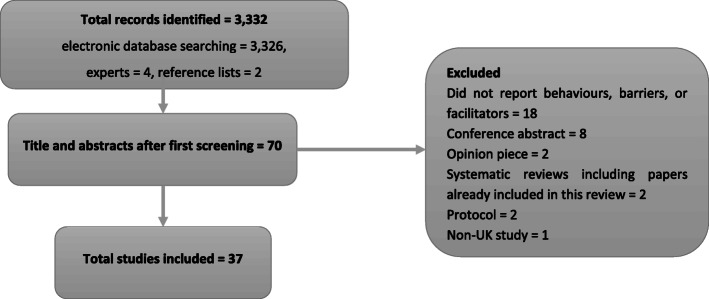
Flow of information through the systematic review

Thirty-seven studies met the inclusion criteria (see Fig. 3). The majority were conducted in primary care (*n* = 28) and collected data from patients (*n* = 25). Table 1 provides a summary of the setting, participants and behaviours investigated. Study quality details are presented in Additional file 6.

**Table 1:** Summary of study characteristics (max *n* = 37 studies)

Setting	
Primary care	28
Community	7
Primary & community	2
Participants	
Patients	23
HCPs	6
Patients & HCPs	2
HCPs & practice managers	2
HCPs, practice managers & commissioners	3
Commissioners	1
Studies reporting influences on these behaviours (total sample size^a^)	
HCPs invite patients for NHS Health Check	4 (171)
Patients attend NHS Health Check	16 (56,909)
HCPs deliver NHS Health Check	18 (10,604)
HCPs refer patients to relevant service	3 (909)
Patients attend referral	1 (483)
Patients change behaviour following NHS Health Check	15 (5755)
Patients attend repeat NHS Health Check	1 (27)
HCPs record NHS Health Check data	2 (2907)
Commissioners synthesise and report programme data	0

^a^Calculated from number of reported participants in each study (seven studies collected national routine data on attendance behaviour only (min 12,000, max > 8,000,000 are not included in this section)

Table 2 describes the setting, participants, behaviour and data collection method for individual studies.

**Table 2:** Individual study characteristics

Reference	Participants	Target behaviour(s)	Data collection method
Primary care			
Alageel, S., M.C. Gulliford, L. McDermott, and A.J. Wright, Implementing multiple health behaviour change interventions for cardiovascular risk reduction in primary care: a qualitative study. BMC family practice. 2018;19:(1): 171 [17].	30 Primary care HCPs	Invite Deliver Refer Patient behaviour change	Qualitative face to face interview
Baker, C., E.A. Loughren, D. Crone, and N. Kallfa, Patients’ perceptions of a NHS Health Check in the primary care setting. Quality in Primary Care. 2014;22:(5): 232–7 [18].	1011 patients who had attended an NHS Health Check	Attend Deliver Patient behaviour change	Postal survey gathering qualitative and quantitative data
Boase, S., D. Mason, S. Sutton, and S. Cohn, Tinkering and tailoring individual consultations: how practice nurses try to make cardiovascular risk communication meaningful. Journal of Clinical Nursing. 2012;21:(17–18): 2590–8 [19].	28 nurses	Deliver	Focus groups and interviews
Chatterjee, R., T. Chapman, M.G. Brannan, and J. Varney, GPs’ knowledge, use, and confidence in national physical activity and health guidelines and tools: a questionnaire-based survey of general practice in England. British Journal of General Practice. 2017;67:(663): e668-e675 [20].	1013 GPs	Deliver	Online quantitative survey
Cook, E.J., C. Sharp, G. Randhawa, A. Guppy, R. Gangotra, and J. Cox, Who uses NHS health checks? Investigating the impact of ethnicity and gender and method of invitation on uptake of NHS health checks. International Journal for Equity in Health. 2016;15:(13) [21]	50,485 patients invited for NHS Health Checks	Attend	Data extracted from routinely collected data on NHS Health Checks
Dalton, A.R., A. Bottle, C. Okoro, A. Majeed, and C. Millett, Uptake of the NHS Health Checks programme in a deprived, culturally diverse setting: cross-sectional study. Journal of Public Health. 2011;33:(3): 422–9 [22].	57,240 patients invited for NHS Health Checks	Behaviour only	Data extracted from routinely collected data on NHS Health Checks
Ellis, N., C. Gidlow, L. Cowap, J. Randall, Z. Iqbal, and J. Kumar, A qualitative investigation of non-response in NHS health checks. Archives of Public Health. 2015;73:(1): 14 [23].	41 NHS Health Check non-attenders	Attend	Interviews
Greaves C, Gillison F, Stathi A, Bennett P, Reddy P, Dunbar J, et al. Waste the waist: a pilot randomised controlled trial of a primary care based intervention to support lifestyle change in people with high cardiovascular risk. International Journal of Behavioral Nutrition & Physical Activity. 2015;12:1 [24].	54 patients	Behaviour only	Patient records
Honey, S., L.D. Bryant, J. Murray, K. Hill, and A. House, Differences in the perceived role of the healthcare provider in delivering vascular health checks: a Q methodology study. BMC Family Practice. 2013;14:(172) [25].	52 primary HCPs	Deliver Patient behaviour change	Q sort task” participants rank a set of predefined attitudinal statements. The sorting patterns of the participants are analysed using by-person correlation and factor analytic techniques to identify distinct ‘clusters of likemindedness’
Honey, S., K. Hill, J. Murray, C. Craigs, and A. House, Patients’ responses to the communication of vascular risk in primary care: a qualitative study. Primary Health Care Research & Development. 2015;16:(1): 61–70 [26].	37 patients at increased risk of CVD	Attend Deliver Patient behaviour change	Qualitative interview
Ismail, H. and K. Atkin, The NHS Health Check programme: insights from a qualitative study of patients. Health Expectations. 2016;19:(2): 345–55 [27].	45 patients 12 month-post NHS Health Check	Attend Deliver Patient behaviour change	Qualitative interview
Ismail, H. and S. Kelly, Lessons learned from England’s Health Checks Programme: using qualitative research to identify and share best practice. BMC Family Practice. 2015;16:(144) [28].	58 staff involved all levels of the delivery of NHS Health Checks	Invite Attend Deliver Patient behaviour change	Qualitative interview
Jenkinson, C.E., A. Asprey, C.E. Clark, and S.H. Richards, Patients’ willingness to attend the NHS cardiovascular health checks in primary care: a qualitative interview study. BMC Family Practice. 2015;16:(33) [29].	17 attendees and 10 non-attendees.	Attend Patient behaviour change Attend repeat	Qualitative interview
Kirby, M. and I. Machen, Impact on clinical practice of the Joint British Societies’ cardiovascular risk assessment tools. International Journal of Clinical Practice. 2009;63:(12): 1683–92 [30].	825 GPs	Deliver Patient behaviour change	Online survey
Krska, J., R. du Plessis, and H. Chellaswamy, Implementation of NHS Health Checks in general practice: variation in delivery between practices and practitioners. Primary Health Care Research & Development. 2016;17:(4): 385–92 [31].	2892 patients	Deliver Record	Patient electronic records
Krska, J., R. du Plessis, and H. Chellaswamy, Views of practice managers and general practitioners on implementing NHS Health Checks. Primary Health Care Research & Development. 2016;17:(2): 198–205 [32].	43 GPs and 40 practice managers	Invite Attend Deliver	Postal survey
McDermott, L., A.J. Wright, V. Cornelius, C. Burgess, A.S. Forster, M. Ashworth, et al., Enhanced invitation methods and uptake of health checks in primary care: randomised controlled trial and cohort study using electronic health records. Health Technology Assessment (Winchester, England). 2016;20:(84): 1–92 [33].	12,052 patients invited for NHS Health Check	Influences on behaviour not reported	Routinely collected data
McNaughton, R.J. and J. Shucksmith, Reasons for (non)compliance with intervention following identification of ‘high-risk’ status in the NHS Health Check programme. Journal of Public Health. 2015;37:(2): 218–25 [34].	29 patients who had undergone NHS Health Check and identified as having increased CVD risk	Patient behaviour change	Interview
Murray, K.A., D.J. Murphy, S.J. Clements, A. Brown, and S.B. Connolly, Comparison of uptake and predictors of adherence in primary and secondary prevention of cardiovascular disease in a community-based cardiovascular prevention programme (MyAction Westminster). Journal of Public Health. 2014;36:(4): 644–50 [35].	483 patients identified at high risk CVD in an NHS Health Check and were referred to a CVD prevention programme	Attend referral	Uptake rate was defined as attendance at the initial assessment; hospital anxiety and depression scale (HADS), health-related quality of life (HRQoL) was assessed with the EuroQol Group 5-Dimension Self-Report Questionnaire score and the EuroQol Visual Analogue Scale of current health status (EQVAS); Brief Illness Perceptions Questionnaire.
Riley, R., N. Coghill, A. Montgomery, G. Feder, and J. Horwood, Experiences of patients and healthcare professionals of NHS cardiovascular health checks: a qualitative study. Journal of Public Health. 2016;38:(3): 543–551 [36].	28 patients and 16 HCPs	Attend Deliver Patient behaviour change	Interviews
Riley, V.A., C. Gidlow, and N.J. Ellis, Uptake of NHS health check: issues in monitoring. Primary Health Care Research & Development. 2018;1–4 [37].	15 commissioners	Record	Interviews
Robson, J., I. Dostal, V. Madurasinghe, A. Sheikh, S. Hull, K. Boomla, et al., NHS Health Check comorbidity and management: an observational matched study in primary care.[Erratum appears in Br J Gen Pract. 2017 Mar;67(656):112; PMID: 28232346]. British Journal of General Practice. 2017;67:(655): e86-e93 [38].	252,259 patients invited for NHS Health Checks in 3 CCGs in East London	Only non-modifiable influences on behaviour reported	Routinely collected data
Burgess, C., A.J. Wright, A.S. Forster, H. Dodhia, J. Miller, F. Fuller, et al., Influences on individuals’ decisions to take up the offer of a health check: a qualitative study. Health Expectations. 2015;18:(6): 2437–2448 [39].	27 patients invited for NHS Health Check	Attend	Interview
Chipchase, L., J. Waterall, and P. Hill, Understanding how the NHS Health Check works in practice. Practice Nursing. 2013;24:(1): 24–29 [40].	10 patients who had undergone NHS Health Checks	Attend Patient behaviour change	Interview
Shaw, R.L., H. Lowe, C. Holland, H. Pattison, and R. Cooke, GPs’ perspectives on managing the NHS Health Check in primary care: a qualitative evaluation of implementation in one area of England. BMJ Open. 2016;6:(7): e010951 [41].	9 GPs	Deliver Refer	Interview
Shaw, R.L., H.M. Pattison, C. Holland, and R. Cooke, Be SMART: examining the experience of implementing the NHS Health Check in UK primary care. BMC Family Practice. 2015;16:(1) [42].	23 Patients and 31 HCPs	Deliver Patient behaviour change	Interview
Attwood, S., K. Morton, and S. Sutton, Exploring equity in uptake of the NHS Health Check and a nested physical activity intervention trial. Journal of Public Health. 2016;38:(3): 560–568 [43].	1165 patients invited for NHS Health Check	Behaviour only	Routinely collected data
Artac, M., A.R.H. Dalton, H. Babu, S. Bates, C. Millett, and A. Majeed, Primary care and population factors associated with NHS Health Check coverage: a national cross-sectional study. Journal of Public Health. 2013;35:(3): 431–439 [44].	40,112 patients invited for NHS Health Check	Behaviour only	Electronic medical records of general practices in Hammersmith and Fulham, London
Primary and community care			
Usher-Smith, J.A., E. Harte, C. MacLure, A. Martin, C.L. Saunders, C. Meads, et al., Patient experience of NHS health checks: a systematic review and qualitative synthesis. BMJ Open. 2017;7:(8): e017169 [45].	20 studies reporting Patient experience of NHS health checks (3497 participants, number of participants not reported for 1 study)”	Attend Deliver Patient behaviour change	Secondary data synthesis
Mills, K., E. Harte, A. Martin, C. MacLure, S.J. Griffin, J. Mant, et al., Views of commissioners, managers and healthcare professionals on the NHS Health Check programme: a systematic review. BMJ Open. 2017;7:(11): e018606 [46].	15 papers reporting views of Commissioners, managers and HCPs (870 participants)	Attend Deliver Refer	Secondary data synthesis
Community care			
Mason, A., D. Liu, L. Marks, H. Davis, D. Hunter, L.M. Jehu, et al., Local authority commissioning of NHS Health Checks: A regression analysis of the first 3 years. Health Policy. 2018;122:(9): 1035–1042 [47].	HPCs and > 8,000,000 patients	Invite	Routinely collected national data
McNaughton, R.J., N.T. Oswald, J.S. Shucksmith, P.J. Heywood, and P.S. Watson, Making a success of providing NHS Health Checks in community pharmacies across the Tees Valley: a qualitative study. BMC Health Services Research. 2011;11:(222) [48].	20 (10 in primary care including DPH, project manager, clinical lead, public health nurses, pharmacy advisor, community services manager, a professional executive committee member and IT database developer; 8 pharmacists, two Local Pharmaceutical Committee representatives)	Attend Deliver	Interviews
Penn, L., A. Rodrigues, A. Haste, M.M. Marques, K. Budig, K. Sainsbury, et al., NHS Diabetes Prevention Programme in England: formative evaluation of the programme in early phase implementation. BMJ Open. 2018;8:(2): e019467 [49].	20 patients referred to NHS Diabetes Prevention Programme following NHS Health Check	Patient behaviour change	Interviews and focus groups
Perry, C., M. Thurston, S. Alford, J. Cushing, and L. Panter, The NHS health check programme in England: a qualitative study. Health Promotion International. 2016;31:(1): 106–15 [50].	36 patients undergoing NHS Health Checks	Attend Deliver Patient behaviour change	Interviews and focus groups
Roberts, D.J. and V.C. de Souza, A venue-based analysis of the reach of a targeted outreach service to deliver opportunistic community NHS Health Checks to ‘hard-to-reach’ groups. Public Health. 2016;137:(176–81) [51].	3849 patients who underwent opportunistic outreach NHS Health Checks	Behaviour only	Routinely collected data for an outreach service in Buckinghamshire.
Taylor, J., J. Krska, and A. Mackridge, A community pharmacy-based cardiovascular screening service: views of service users and the public. International Journal of Pharmacy Practice. 2012;20:(5): 277–84 [52].	259 patients who had undergone NHS Health Check, and 261 non-service users	Attend	Questionnaire
Visram, S., S.M. Carr, and L. Geddes, Can lay health trainers increase uptake of NHS Health Checks in hard-to-reach populations? A mixed-method pilot evaluation. Journal of Public Health. 2015;37:(2): 226–33 [53].	181 patients	Attend	Questionnaire

* Reported data on behaviour only (not influences on behaviour)

### Systems map of NHS health check behaviours

**Fig. 4:**
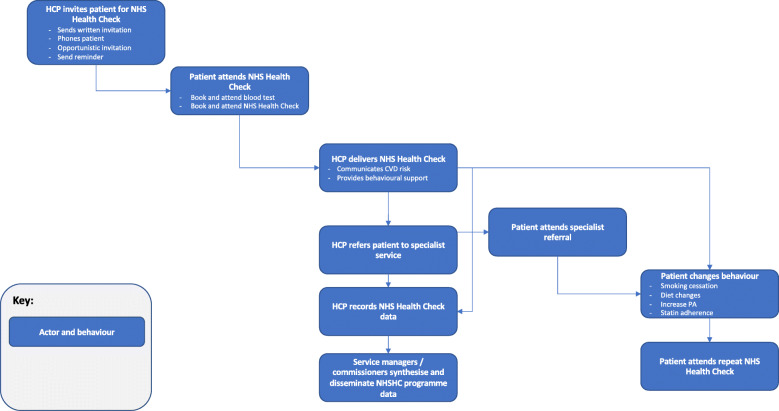
Behavioural systems map of NHSCHC behaviours

Behaviours reported in the systematic review of barriers to and facilitators of NHS Health Check-related behaviours are provided in the behavioural systems map Fig. 4. Studies identified in the systematic review typically focussed on barriers to and facilitators of HCPs delivering NHS Health Checks (*n* = 18 studies), patients attending NHS Health Checks (*n* = 16 studies) and patients changing behaviour following NHS Health Checks (*n* = 15 studies). The map is divided into behaviours occurring at five sequential time periods: i) HCPs inviting patients to attend NHS Health Checks; ii) patients attending NHS Health Checks; iii) HCPs delivering NHS Health Checks; HCPs recording NHS Health Check data; service managers and/or commissioners synthesising and disseminating NHS Health Check data; iv) patients attending specialist referral; v) patient changing CVD risk-related behaviours; patients attending repeat NHS Health Check.

### Barriers to and facilitators of NHS health check behaviours

Domains and themes identified as relevant to each NHS Health Check behaviour is provided in Table 3. Barriers, facilitators or both barriers and facilitators for each behaviour is summarised in Table 4.

**Table 3:** Frequency of domains and all themes related to each identified behaviour

Theme (number of studies) Domain	Frequency (number of studies)	Elaboration (number of themes)	Evidence of both barriers and facilitators
HCP issuing invitation (*n* = 4)			
Environmental context and resources	4	2	n
Memory attention and decision processes	1	1	n
Patient attending (*n* = 16)			
Environmental context and resources	12	6	y
Social influences	8	5	y
Beliefs about consequences	7	6	y
Emotion	6	3	n
Knowledge	6	1	n
Memory attention and decision processes	4	2	y
Social professional role and identity	3	3	y
Beliefs about capabilities	3	2	y
Cognitive and interpersonal skills	1	1	n
HCP Delivering NHS Health Check (*n* = 18)			
Environmental context and resources	13	8	y
Beliefs about consequences	12	5	y
Social professional role and identity	9	3	y
Cognitive and interpersonal skills	9	2	y
Social influences	8	4	y
Optimism	5	1	y
Memory attention and decision processes	4	3	y
Knowledge	4	2	y
Beliefs about capabilities	4	1	y
Emotion	3	2	y
Goals	1	1	y
Intentions	1	1	n
Behavioural regulation	1	1	n
Reinforcement	1	1	n
HCP making referral after NHS Health Check (*n* = 3)			
Environmental context and resources	3	1	n
Social influences	1	1	n
Patient attend referral (*n* = 1)			
Beliefs about consequences	1	1	n
Patient behaviour change (*n* = 15)			
Knowledge	10	2	y
Intentions	8	2	y
Environmental context and resources	6	5	y
Social influences	5	2	n
Beliefs about capabilities	4	1	n
Beliefs about consequences	3	4	n
Social professional role and identity	2	2	y
Optimism	2	1	n
Patient attend repeat NHS Health Check (*n* = 1)			
Intentions	1	1	n
HCP record programme data (*n* = 2)			
Environmental context and resources	1	3	y^a^
Behavioural regulation	1	1	y
Social professional role and identity	1	1	y

^a^This is a summary of *all* themes and domain coding for barriers and facilitators may differ to Table 4 which are colour coded according to domains and or themes within domains identified as *key*. For example, Environmental context and resources was identified as a domain relevant HCP recording programme data and contained three themes (a mixture of barriers and facilitators). However, the theme identified as key was a barrier and is presented as such in Table 4

**Table 4:**
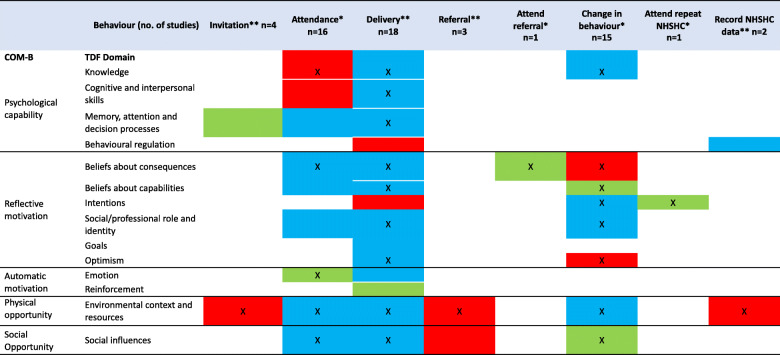
Classification of TDF domains (all themes) as Barriers, Facilitators, or Both across behaviours

* Patient behaviour

** HCP behaviour

X Key theme

Red cell = Barrier (all themes within the domain are barriers)

Green cell = Facilitator (all themes within the domain are facilitators)

Blue cell = Both (themes within the domain are a mixture of barriers and facilitator)

White cells = TDF domain not linked to behaviour

#### Key barriers to and facilitators of NHS health check behaviours

**Fig. 5:**
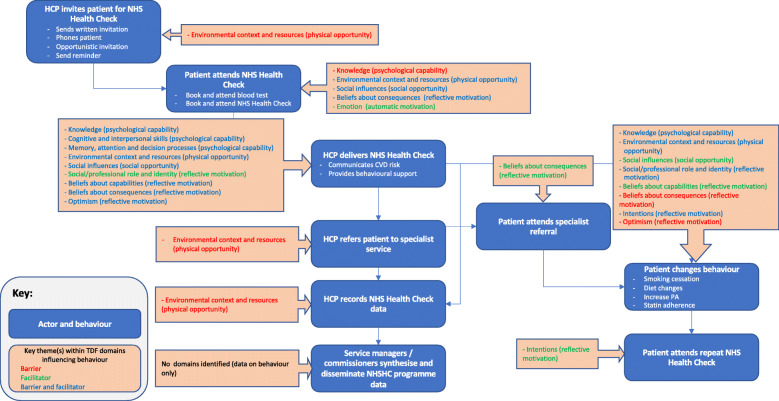
Summary of key influences on behaviours

Domains and themes identified as key in influencing NHS Heath Check behaviours are summarised for each behaviour below and in Fig. 5 and Table 5 along with illustrative examples.

1. *HCPs inviting patients to attend NHS Health Checks***.** Domains related to this behaviour were identified in four studies. The key domain was ‘environmental context and resources.’ The key theme within this domain was: Difficulty identifying eligible patients from records as a barrier to issuing invitations.
2. *Patients attending NHS Health Checks.* Domains related to this behaviour were identified in 16 studies. Five key domains were identified:
  i. ‘Knowledge’ - a lack of understanding of CVD risk and purpose of NHS Health Checks – coded as a barrier.
  ii. ‘Environmental context and resources’ – i) timing and location of NHS Health Checks influenced attendance (some perceived timing and location to be convenient, others did not); ii) conducting NHS Health Checks in pharmacies. Both themes were coded as both barriers and facilitators.
  iii. ‘Social influences’ – i) having a family history of illness was coded as both a barrier and facilitator as it discouraged some from attending and encouraged others to attend; ii) the impact of interactions with GP with which they did not have a good relationship and being told to change was coded as a barrier.
  iv. ‘Beliefs about consequences’ – i) There were a range of views on the extent to which NHS Health Checks were perceived as beneficial for early detection, this was coded as both barrier and facilitator reflecting opposing views within this theme; ii) NHS Health Checks provided the opportunity to be proactive about health - this was coded as a facilitator.
  v. ‘Emotion’ – i) anxiety at receiving high risk result; ii) reassurance as a motivation to attend. Both themes were coded as facilitators as anxiety and reassurance encouraged attendance.
3. *HCPs delivering NHS Health Checks***.** Nine key domains were identified across 18 studies:
  i. ‘Knowledge’ – i) HCPs perceptions of patients understanding of CVD risk was coded as both barrier and facilitator as some HCPs considered that patients understood their risk, but this view was not shared by all; ii) HCPs lack of familiarity with guidelines and associated tools which was coded as a barrier.
  ii. ‘Cognitive and interpersonal skills’ - HCPs perceived the need for i) training to deliver behavioural support and, ii) to communicate risk. The former was coded as both barrier and facilitator as some but not all perceived the need for training to improve behavioural support skills, the latter was coded as a barrier as all reported the need to improve risk communication skills.
  iii. ‘Memory, attention and decision processes’ - HCPs had different views on whether behavioural intervention should be offered before pharmacological intervention to reduce CVD risk so this was coded as a both a barrier and facilitator.
  iv. ‘Environmental context and resources’ – i) availability of resources and time to deliver NHS Health Checks was coded as both barrier and facilitator reflecting different access to resources and different amounts of time to deliver NHS Health Checks; ii) lack of appropriate space to deliver NHS Health Checks was coded as a barrier; iii) having computer systems which support delivery of NHS Health Checks was coded as both barrier and facilitator.
  v. ‘Social influences’ – taking account of patients’ social context when providing behavioural support was coded as both barrier and facilitator as some but not all HCPs considered patients’ social context.
  vi. ‘Social/professional role and identity’ – i) some but not all HCPs thought there was role clarity within teams when delivering NHS Health Checks, so this was coded as a barrier and facilitator; ii) all pharmacy staff reported that delivering NHS Health Checks positively promoted diversification of their professional role so this was coded as a facilitator.
  vii. ‘Beliefs about consequences’ – i) holding the belief that NHS Health Checks are beneficial in terms of preventive healthcare; ii) believing appropriately framing messages is important. Both themes were coded as both barrier and facilitator reflecting opposing beliefs within each theme.
  viii. ‘Beliefs about capabilities’ - HCPs varied in their level of confidence to discuss and initiate behaviour change in patients, so this was coded as both a barrier and facilitator.
  ix. ‘Optimism’ - HCPs are varyingly optimistic about whether patients will change their behaviour after the NHS Health Check, this was coded as both a barrier and facilitator.
4. *HCP referral to specialist service.* The key domain, ‘environmental context and resources’ was identified in three studies and contained the theme, lack of funded services to refer patients to. This was coded as a barrier.
5. *Patients attending specialist referral.* One key domain, ‘beliefs about consequences’ was identified in one study and contained the theme, holding the belief that regular attendance at referral appointments would help to reduce CVD risk. This was coded as a facilitator.
6. *Patients changing CVD risk-related behaviours.* Eight key domains were identified across 15 studies:
  i. ‘Knowledge’ - Patients’ understanding of CVD risk was coded as both barrier and facilitator as some HCPs considered that patients understood their risk, but this view was not shared by all.
  ii. ‘Environmental context and resources’ – i) time and cost as a barrier to patients changing behaviours related to CVD risk - this was coded as both barrier and facilitator as some reported not having time or funds to change behaviour whilst others reported sufficient time and funds; ii) HCPs perceived there to be greater adherence to behavioural support when CVD risk was communicated using electronic calculators than paper risk charts – this was coded as a facilitator
  iii. ‘Social influences’ – support from family and friends was reported by patients as a facilitator of behaviour change to reduce CVD risk.
  iv. ‘Social/professional role and identity’ – Patients perceived the role of the HCP to influence their engagement with changing behaviour to reduce CVD risk, as there were differing views of which role would most effectively encourage patients to change (e.g., GPs, practice nurses, community HCPs) this was coded as both barrier and facilitator.
  v. ‘Beliefs about capabilities’ – Patients reported the belief that they were able to change their behaviour to reduce CVD risk if changes were small and sustainable – this was coded as a facilitator.
  vi. ‘Beliefs about consequences’ – i) patients reported contradictory guidelines on healthy behaviours was a barrier to behaviour change; ii) related to the previous theme, patients often reported inaccurate beliefs of what constitutes a healthy behaviour, e.g. binge drinking was not harmful if done infrequently – this was coded as a barrier.
  vii. ‘Intentions’ – Patients reported different views on whether the NHS Health Check was a ‘wake-up call’ to change their behaviour – this was coded as both a barrier and facilitator.
  viii. ‘Optimism’ – Patients’ fatalistic beliefs about their health (i.e. there was nothing they could do to change whether they would experience a cardiovascular event) was a barrier to change.
7. *Patients attending repeat NHS Health Check.* One key domain was identified in one study. The domain ‘Intentions’ contained the theme – intention to attend a future NHS Health Check. This was coded as a facilitator as, where it was discussed, patients reported the intention to attend a repeat check.
8. *HCPs recording NHS Health Check data.* One key domain was identified across two studies. ‘Environmental context and resources’ – HCPs reported that multiple methods of invitation to NHS Health Checks was a barrier to accuracy of reporting relevant data.

**Table 5:** Key domains, themes and illustrative quotes

TDF domain (COM-B)	Theme	*Illustrative extract*
Invitation (HCP)		
Environmental context and resources (physical opportunity)	Difficulty identifying eligible patients from records	*“I print off the list of patients from and I trawl through them, looking, making sure that they’re all within that bracket, because there’s some that are underage (under 40) and some that are over the age (over 74), so it’s a pain, filtering those patients out, 19-year-olds and 90-odd-year-olds on there”* [25]
Attendance (patient)		
Knowledge (psychological capability)	Lack of understanding of CVD risk and purpose of NHS HC	*“I’d had cholesterol tests, I’d had weight and height, I’d had more or less the whole health check very recently. So, I phoned up my GP and said ‘Look I’ve just had this’.*. *. I want to make sure that it’s worth my time and the GP’s time and the NHS time to do it.”* [39]
Environmental context and resources (physical opportunity)	Timing and location of NHS HCs increased attendance	*“I mean we have the healthcare assistants are here at 8:30, so they can have early bloods done before they go to work, and the nurse can see them before they go to work, so we’re trying to offer those facilities to people to catch them.”* [28]
		*“It’s very difficult for me to (go to the appointment) and hold on to a nine-to-five job. It means I have to take personal time off from my employer to do this. They don’t give you an option where you can go in the evening. I would have to take it off as annual leave, and do it in my own personal time.”* [39]
	Conducting NHS HCs in pharmacies	*“Oh, very easy, I mean I just walked in there and booked myself in.*. *. I think I’d gone in the morning and I’d booked in for early afternoon and then went to do some shopping and went back.”* [39]
		*“Some [pharmacies] are really struggling because they are out of town and in locations close to GP practices - which is where the pharmacies get a lot of their business from.”* [48]
Social influences (social opportunity)	Family history of illness	*“*. *. family history is obviously, you know, a huge determinant of various things. OK not completely conclusive, but you know, law of averages, I thought I’m probably OK. So, it just slipped and then I never took up on it.”* [39]
		*“I suppose the fact that my father died relatively young of a heart attack, probably made me fairly aware of the need to try and be healthy.*. *. I suppose I was thinking everybody needs to be careful when they get to their mid-fifties.”* [39]
	Interactions with GP/ being told to change	*“I didn’t want to find out I had more medical problems, I have epilepsy. And I don’t need a doctor to tell me I need to stop smoking and lose weight.”* [39]
Beliefs about consequences (reflective motivation)	NHS HCs not always perceived beneficial for early detection	*Virtually all of the service users (96; 99%) felt that the screening had been of benefit to them, with 29 (30.5%) stating that the screening had identified problems of which they were previously unaware* [52]*.* *“I don’t think there is an awful lot of value. I think you’ll pick up a few people a little bit earlier. Now whether that’s worth the cost, obviously it’s great for those individual patients, whether that’s worth the cost of running a programme like this. I’d be amazed if it was.* “ [46]
	NHS HCs provided opportunity to be proactive about health	*“I just think it makes you more aware and if you do have any problems you have the chance to actually, you know, be proactive to it rather than reactive when maybe things are a bit too late.”* [29]
Emotion (automatic motivation)	Anxiety at receiving high risk result	*“I got a letter from the doctor’s saying ‘as you are at a high risk of a stroke or heart attack’...well I nearly died, and I thought ‘well what have my results come up as?’ And so of course I made an appointment and I went on.* “ [26]
	Reassurance as a motivation to attend	*“Well in one way it’s a reassurance if there’s nothing wrong. It’s an opportunity to be reminded that you should take care of your health.”* [39]
Delivering NHS HC (HCP)		
Knowledge (psychological capability)	HCPs perceptions of patients understanding of CVD risk	*Several GPs made the point that the important message to convey to patients was to reduce their risk factors and the accuracy of the figures was less important than this central message* [30]*.*
		*Participants believed that most patients knew what constituted health-enhancing behaviours and the impact of risk behaviours on their health. Therefore, HCPs perceived little need to explain the importance of changing behaviour to patients* [17]*.*
	HCPs familiarity with guidelines and associated tools	*59% had either not heard of (30%) or were very unfamiliar with CMO’s PA guidelines* [20]*.*
Cognitive and interpersonal skills (psychological capability)	HCPs perceived need for training to deliver behavioural support	*“[Training] would be good. As I say, we just learnt from our healthcare assistant what to do; basically, it was like kind of on the job training… It would be nice to understand it in depth more, wouldn’t it?”* [28] *GPs reported a lack of specific training to implement behaviour change interventions, but some GPs believed that they did not need special training to implement behaviour change interventions. It was viewed as being intrinsic to their medical training* [17]*.*
	HCP training to communicate risk	*“You’ll have all the staff who may not be aware of what harm they could be doing…So I think the person would have to have the skill to know this patient doesn’t wish to know.”* [19]
Memory attention decision processes (psychological capability	Behavioural intervention before pharmacological intervention	*Health professionals thought that patients were more likely to take medication rather than make lifestyle changes to reduce risk, as this was easier* [30]*.* *There was a view that as lifestyle change is possible, opting for medication as a first line treatment is not the best strategy* [25]*.*
Environmental context and resources (physical opportunity)	Limited time/ resources to deliver NHS HC	*The 10-min consultation was considered too short to perform the risk assessment, give patient-centred lifestyle advice, and fully explain any prescribed medication* [19]*.*
	Appropriate space to deliver NHS HC	*“I don’t think you come across very professional when you’re sitting in a kitchen and all huddled round and all on top of each other. And it’s not very nice for the patients, because…quite personal information.”* [46]
	Computer systems supporting NHS HC delivery	*Difficulties with information technology and computer software were mentioned in over half of the studies; 39% of practice managers in one study reported difficulties with the clinical system, software or errors in the existing data* [32]*.*
Social influences (social opportunity)	Taking account of patients’ social context	*Nurses considered it important to understand people’s social context, so that conversations about risk could always be individually appropriate* [19]*.*
		*Ten felt that the nurse/HCA failed to provide tailored advice that took into account their individual capabilities… or their particular circumstances such as availability of recreational spaces and childcare issues* [27]*.*
Social/professional role and identity (reflective motivation)	Role clarity in delivering NHS HCs	*Some participants felt advocating for behaviour change was an essential part of their job, which by itself was an enough of an incentive* [17]*.*
	Diversification of pharmacy staff role	*The focus was on the benefits of delivering NHS Health Checks in pharmacies, with all feeling it offered immense job satisfaction, promoted the image of the pharmacy and provided a good opportunity for staff development* [46]*.*
Beliefs about consequences (reflective motivation)	Belief that NHS HCs were beneficial in terms of preventive healthcare	*“I think it’s a very good idea. We have a very high proportion of our patients who suffer with diabetes, almost 10% of our patients are diabetic so I thought this was an excellent opportunity to screen those earlier and pick them up and then you know be able to do something about it, you know, lifestyle management.”* [41] *“I think really this is mass screening and there’s not a great deal of proof behind it…. Not entirely convinced with being told we have to offer a check to everyone.”* [46]
	Message framing	*A frequently reported style was downplaying. Here health care professionals appeared to downplay the individual’s high-risk score by using phrases such as, ‘it is only slightly higher’ if the risk score was, for example, between 20 and 25%. As a consequence, some patients concluded that the risk was not particularly significant* [26]*.*
		*One strategy that many described was to concentrate on risk reduction without actually talking to a patient about risk scores explicitly at all. Thus, lifestyle advice and behaviour change were frequently raised not in terms of a patient’s cardiovascular risk* per se*, but in more general terms around the idea of maintaining or improving health* [19]*.*
Beliefs about capabilities (reflective motivation)	HCP confidence in discussing and initiating behaviour change	*There is a strong belief in the ability of healthcare professionals to motivate patients to change their lifestyle* [25]*.* *Pharmacy staff had also required a lot more training than initially anticipated and, even after being given this support, lacked confidence in delivering the new service* [48]*.*
Optimism (reflective motivation)	HCPs varyingly optimistic about patient behaviour change after NHS HC	*There was a clear rejection of pessimism about the possibility of lifestyle change* [25]*.*
		*Even if you access them, even if you find out that they’re a really high-risk score then getting these people to take on board you know the lifestyle changes, changes to their diet, exercising more. It’s very difficult to get them to take those changes on* [28]*.*
Referral to specialist service (HCP)		
Environmental context and resources (physical opportunity)	Lack of funded services to refer patients to	*Healthcare professionals expressed the importance of making referrals to external lifestyle services to support patients through the behaviour change process, but these services had difficulties with long waiting lists, budget cuts causing the discontinuation of some services and were not always offered at times that suited the working population* [17]*.*
Attending referral (patient)		
Beliefs about consequences (reflective motivation)	Regular attendance is important	*The belief that attending regular appointments would reduce CVD risk predicted adherence to the programme* [35]*.*
Patient changing behaviour (patient)		
Knowledge (psychological capability)	Patient understanding of CVD risk and its implications after NHS HC	*Two-thirds of patients ...rating their understanding of the CVD risk score highly (4 or above on a scale of 1 to 5, 5 indicating a high level of understanding)* [18]*.*
		*“The conclusion was I have a 6% chance of getting heart disease, which on one hand sounds good because 6 people out of 100, but then if I’m one of those 6 … so I feel very unclear about it. I thought, well how close to 10 is 6?”* [40]
Environmental context and resources (physical opportunity)	Time and cost as a barrier to adherence	*A number of patients, especially from lower socio-economic groups, encountered barriers in adopting healthy eating, citing the cost of eating fresh fruit and vegetables* [27]*.*
		*Social and material factors were not seen as real impediments to lifestyle change* [25]*.*
	Adherence to behavioural support influenced by mode of communication of risk	*A higher proportion [of HCPs] thought that most patients complied with advice received during a consultation with the electronic calculator than the paper risk charts* [30]*.*
Social influences (social opportunity)	Support from others to change	*Participants also spoke about the importance of family and friends in supporting the changes they made. The significance of family networks, particularly immediate family relatives, reveals that social ties are an aspect of people’s everyday lives that could enable or constrain desired changes in behaviour* [50]*.*
Social professional role and identity (reflective motivation)	Patient engagement is influenced by HCP role	*In some interviews, participants discussed how different clinicians influenced the success of behaviour change interventions. Some thought that if the intervention was delivered by a GP it would have a bigger influence on patients. Others argued that patients might be more open and engaged with interventions delivered by nurses and HCAs, due to the ease of the relationship. One manager thought that patients would be more open in community settings rather than with their healthcare provider* [17]*.*
Beliefs about capabilities (reflective motivation)	Changes perceived to be achievable	*For many participants, making small and sustainable changes to their diet by consuming less salt and fat was achievable, as long as it did not cause too much disruption to their daily routines* [34]*.*
Beliefs about consequences (reflective motivation)	Contradictory guidelines	*People showed reluctance to make changes to their lifestyle, noting that any guidance they were given was likely to be subject to change…. stating that previous guidance about healthy eating suggested that the consumption of eggs should be restricted; then the reverse was promoted* [26]*.*
	Perceptions of what constitutes healthy behaviour	*A number of people in this group reported drinking well in excess of the recommended units of alcohol but were unconvinced about how many units were considered harmful:* *Pt E: If I go out on a Saturday night, I’ll have 10 pints.* *Int: Right, and do you see this as a risk to your health?* *Pt E: No because I am only having 10 pints a fortnight – one must balance the other* [26]*.*
Intentions (reflective motivation)	NHS HCs as a ‘wake-up call’	*It’s really good. It makes you aware of what problems are around. What you can get and that. It is really good. It teaches you. it’s an eye-opener for people who would want to do things properly* [45]*.* *Several people chose not to try and lower their cardiovascular risk because they believed death from a heart attack would be preferable to dying from a protracted illness or living into extreme old age: “I am not afraid of death. If I go, I go but I want it to be quick.”* [26]
Optimism (reflective motivation)	Fatalistic views about disease	*“You can be as careful as you want; you can eat as healthily as you want; you can do all the exercise you want and you could still get ill. It is like J’s mother who lived to be 101, smoked like a trooper, never had a cigarette out of her hand and she died of something silly.”* [26]
Attending repeat NHS HC (patient)		
Intention (reflective motivation)	Likelihood of attending future NHS HC	*”I think, well, I eat healthily, you know, and I believe in a healthy lifestyle. I know there are other internal things which could go wrong. But yeah, I would definitely have it again if it came up next week, you know, I didn’t realise it was all about different things.”* [29]
Recording NHS HC data (HCP)		
Environmental context and resources (physical opportunity)	Accuracy of recording is compromised by multiple methods of invitation	*The combination of opportunistic NHS HCs and the delay in time between patients receiving an invitation and attending an NHS HC also caused problems for participants when reporting quarterly data: ‘a health check received doesn’t correlate for a health check offered* [37]*.*

### Signposting to relevant intervention content

Intervention content (intervention types, policy categories and BCTs) which is theoretically linked to identified influences on NHS Health Check behaviours which could be considered for inclusion in intervention design and refinement is suggested in Table 6. Example suggestions for how BCTs could be delivered to target key barriers to and facilitators of NHS Health Check behaviours are provided in Table 7.

**Table 6:** Suggested intervention functions and BCTs to target key themes influencing NHS Health Check behaviours

TDF domain (COM-B)	Theme (barrier, facilitator, both barrier and facilitator)	*Potential intervention functions*^*a*^	*Potential policy categories*	Potential BCTs
HCP issuing invitation				
Environmental context and resources (physical opportunity)	Difficulty identifying eligible patients from records	*Training* *Restriction* *Environmental restructuring* *Enablement*	Guidelines Fiscal measures Regulation Legislation Environmental/ Social planning (not training or restriction) Service provision (not restriction or environmental restructuring)	Social support – practical (advise on, arrange, or provide practical help (e.g. from friends, relatives, colleagues, ‘buddies’ or staff) for performance of the behaviour) Prompts/cues (introduce or define environmental or social stimulus with the purpose of prompting or cueing the behaviour normally occurring at the time or place of performance) Remove aversive stimulus (advise or arrange for the removal of an aversive stimulus to facilitate behaviour change) Restructuring the physical environment (change, or advise to change the physical environment in order to facilitate performance of the wanted behaviour or create barriers to the unwanted behaviour (other than prompts/cues, rewards and punishments) Restructuring the social environment (change, or advise to change the social environment in order to facilitate performance of the wanted behaviour or create barriers to the unwanted behaviour (other than prompts/cues, rewards and punishments) Avoidance/reducing exposure to cues for the behaviour (advise on how to avoid exposure to specific social and contextual/physical cues for the behaviour, including changing daily or weekly routines) Adding objects to the environment (add objects to the environment in order to facilitate performance of the behaviour)
Patient attendance				
Knowledge (psychological capability)	Lack of understanding of CVD risk and purpose of NHS Health Checks	*Education*	Communication/ marketing Guidelines Regulation Legislation Service provision	Biofeedback (provide feedback about the body (e.g. physiological or biochemical state) using an external monitoring device as part of a behaviour change strategy) Instruction on how to perform behaviour (advise or agree on how to perform the behaviour (includes ‘Skills training’)) Information about antecedents (provide information about antecedents) Information about health consequences (provide information (e.g. written, verbal, visual) about health consequences of performing the behaviour) Salience of consequences (use methods specifically designed to emphasise the consequences of performing the behaviour with the aim of making them more memorable (goes beyond informing about consequences)) Information about social and environmental consequences (provide information (e.g. written, verbal, visual) about social and environmental consequences of performing the behaviour)
Environmental context and resources (physical opportunity)	Timing and location of NHS Health Checks increased attendance	*Training* *Restriction* *Environmental restructuring* *Enablement*	Guidelines Fiscal measures Regulation Legislation Environmental/ Social planning (not training or restriction) Service provision (not restriction or environmental restructuring)	Social support – practical (advise on, arrange, or provide practical help (e.g. from friends, relatives, colleagues, ‘buddies’ or staff) for performance of the behaviour) Prompts/cues (introduce or define environmental or social stimulus with the purpose of prompting or cueing the behaviour normally occurring at the time or place of performance) Remove aversive stimulus (advise or arrange for the removal of an aversive stimulus to facilitate behaviour change) Restructuring the physical environment (change, or advise to change the physical environment in order to facilitate performance of the wanted behaviour or create barriers to the unwanted behaviour (other than prompts/cues, rewards and punishments) Restructuring the social environment (change, or advise to change the social environment in order to facilitate performance of the wanted behaviour or create barriers to the unwanted behaviour (other than prompts/cues, rewards and punishments) Avoidance/reducing exposure to cues for the behaviour (advise on how to avoid exposure to specific social and contextual/physical cues for the behaviour, including changing daily or weekly routines) Adding objects to the environment (add objects to the environment in order to facilitate performance of the behaviour)
	Conducting NHS Health Checks in pharmacies	*Training* *Restriction* *Environmental* *restructuring* *Enablement*	Guidelines Fiscal measures Regulation Legislation Environmental/ Social planning (not training or restriction) Service provision (not restriction or environmental restructuring)	Social support – practical (advise on, arrange, or provide practical help (e.g. from friends, relatives, colleagues, ‘buddies’ or staff) for performance of the behaviour) Prompts/cues (introduce or define environmental or social stimulus with the purpose of prompting or cueing the behaviour normally occurring at the time or place of performance) Remove aversive stimulus (advise or arrange for the removal of an aversive stimulus to facilitate behaviour change) Restructuring the physical environment (change, or advise to change the physical environment in order to facilitate performance of the wanted behaviour or create barriers to the unwanted behaviour (other than prompts/cues, rewards and punishments) Restructuring the social environment (change, or advise to change the social environment in order to facilitate performance of the wanted behaviour or create barriers to the unwanted behaviour (other than prompts/cues, rewards and punishments) Avoidance/reducing exposure to cues for the behaviour (advise on how to avoid exposure to specific social and contextual/physical cues for the behaviour, including changing daily or weekly routines) Adding objects to the environment (add objects to the environment in order to facilitate performance of the behaviour)
Social influences (social opportunity)	Family history of illness	*Restriction* *Environmental restructuring* *Modelling* *Enablement*	Communication/ marketing (modelling only) Guidelines (not modelling) Fiscal measures (not modelling or restriction) Regulation (not modelling) Legislation (not modelling) Environmental/ Social planning (not modelling or restriction) Service provision (not restriction or environmental restructuring)	Social support – unspecified (advise on, arrange or provide social support (e.g. from friends, relatives, colleagues,’ buddies’ or staff) or non-contingent praise or reward for performance of the behaviour. It includes encouragement and counselling, but only when it is directed at the behaviour) Social support – practical (advise on, arrange, or provide practical help (e.g. from friends, relatives, colleagues, ‘buddies’ or staff) for performance of the behaviour) Social comparison (draw attention to others’ performance to allow comparison with the person’s own performance) Information about others’ approval (provide information about what other people think about the behaviour. The information clarifies whether others will like, approve or disapprove of what the person is doing or will do) Social reward (arrange verbal or non-verbal reward if and only if there has been eff ort and/or progress in performing the behaviour (includes ‘Positive reinforcement’))
	Interactions with GP/ being told to change	*Restriction* *Environmental restructuring* *Modelling* *Enablement*	Communication/ marketing (modelling only) Guidelines (not modelling) Fiscal measures (not modelling or restriction) Regulation (not modelling) Legislation (not modelling) Environmental/ Social planning (not modelling or restriction) Service provision (not restriction or environmental restructuring)	Social support – unspecified (advise on, arrange or provide social support (e.g. from friends, relatives, colleagues,’ buddies’ or staff) or non-contingent praise or reward for performance of the behaviour. It includes encouragement and counselling, but only when it is directed at the behaviour) Social support – practical (advise on, arrange, or provide practical help (e.g. from friends, relatives, colleagues, ‘buddies’ or staff) for performance of the behaviour) Social comparison (draw attention to others’ performance to allow comparison with the person’s own performance) Information about others’ approval (provide information about what other people think about the behaviour. The information clarifies whether others will like, approve or disapprove of what the person is doing or will do) Social reward (arrange verbal or non-verbal reward if and only if there has been eff ort and/or progress in performing the behaviour (includes ‘Positive reinforcement’))
Beliefs about consequences (reflective motivation)	NHS Health Checks not always perceived beneficial for early detection	*Education* *Persuasion* *Modelling*	Communication/ marketing Guidelines (not modelling) Regulation (not modelling) Legislation (not modelling) Service provision	Information about health consequences (provide information (e.g. written, verbal, visual) about health consequences of performing the behaviour) Salience of consequences (use methods specifically designed to emphasise the consequences of performing the behaviour with the aim of making them more memorable (goes beyond informing about consequences)) Information about social and environmental consequences (provide information (e.g. written, verbal, visual) about social and environmental consequences of performing the behaviour) Anticipated regret (induce or raise awareness of expectations of future regret about performance of the unwanted behaviour) Information about emotional consequences (provide information (e.g. written, verbal, visual) about emotional consequences of performing the behaviour) Pros and cons (advise the person to identify and compare reasons for wanting (pros) and not wanting to (cons) change the behaviour (includes ‘decisional balance’)) Comparative imagining of future outcomes (prompt or advise the imagining and comparing of future outcomes of changed versus unchanged behaviour) Material incentive - behaviour (inform that money, vouchers or other valued objects will be delivered if and only if there has been eff ort and/or progress in performing the behaviour (includes ‘Positive reinforcement’)) Incentive – outcome (inform that a reward will be delivered if and only if there has been eff ort and/or progress in achieving the behavioural outcome (includes ‘positive reinforcement’)) Reward - outcome (arrange for the delivery of a reward if and only if there has been eff ort and/or progress in achieving the behavioural outcome (includes ‘positive reinforcement’))
	NHS Health Checks provided opportunity to be proactive about health	*Education* *Persuasion* *Modelling*	Communication/ marketing Guidelines (not modelling) Regulation (not modelling) Legislation (not modelling) Service provision	Information about health consequences (provide information (e.g. written, verbal, visual) about health consequences of performing the behaviour) Salience of consequences (use methods specifically designed to emphasise the consequences of performing the behaviour with the aim of making them more memorable (goes beyond informing about consequences)) Information about social and environmental consequences (provide information (e.g. written, verbal, visual) about social and environmental consequences of performing the behaviour) Anticipated regret (induce or raise awareness of expectations of future regret about performance of the unwanted behaviour) Information about emotional consequences (provide information (e.g. written, verbal, visual) about emotional consequences of performing the behaviour) Pros and cons (advise the person to identify and compare reasons for wanting (pros) and not wanting to (cons) change the behaviour (includes ‘decisional balance’) Comparative imagining of future outcomes (prompt or advise the imagining and comparing of future outcomes of changed versus unchanged behaviour) Material incentive - behaviour (inform that money, vouchers or other valued objects will be delivered if and only if there has been eff ort and/or progress in performing the behaviour (includes ‘Positive reinforcement’)) Incentive – outcome (inform that a reward will be delivered if and only if there has been eff ort and/or progress in achieving the behavioural outcome (includes ‘positive reinforcement’)) Reward - outcome (arrange for the delivery of a reward if and only if there has been eff ort and/or progress in achieving the behavioural outcome (includes ‘positive reinforcement’))
Emotion (automatic motivation)	Anxiety at receiving high risk result	*Persuasion* *Incentivisation* *Coercion* *Modelling* *Enablement*	Communication/ marketing (not enablement) Guidelines (not modelling) Fiscal measures (not persuasion or modelling) Regulation (not modelling) Legislation (not modelling) Environmental/ Social planning (enablement only) Service provision	Reduce negative emotions (advise on ways of reducing negative emotions to facilitate performance of the behaviour (includes ‘stress management’))
	Reassurance as a motivation to attend	*Persuasion* *Incentivisation* *Coercion* *Modelling* *Enablement*	Communication/ marketing (not enablement) Guidelines (not modelling) Fiscal measures (not persuasion or modelling) Regulation (not modelling) Legislation (not modelling) Environmental/ Social planning (enablement only) Service provision	Reduce negative emotions (advise on ways of reducing negative emotions to facilitate performance of the behaviour (includes ‘stress management’))
HCP delivering NHS Health Check				
Knowledge (psychological capability)	HCPs perceptions of patients understanding of CVD risk	*Education*	Communication/ marketing Guidelines Regulation Legislation Service provision	Biofeedback (provide feedback about the body (e.g. physiological or biochemical state) using an external monitoring device as part of a behaviour change strategy) Instruction on how to perform behaviour (advise or agree on how to perform the behaviour (includes ‘Skills training’)) Information about antecedents (provide information about antecedents) Information about health consequences (provide information (e.g. written, verbal, visual) about health consequences of performing the behaviour) Salience of consequences (use methods specifically designed to emphasise the consequences of performing the behaviour with the aim of making them more memorable (goes beyond informing about consequences)) Information about social and environmental consequences (provide information (e.g. written, verbal, visual) about social and environmental consequences of performing the behaviour)
	HCPs familiarity with guidelines and associated tools	*Education*	Communication/ marketing Guidelines Regulation Legislation Service provision	Biofeedback (provide feedback about the body (e.g. physiological or biochemical state) using an external monitoring device as part of a behaviour change strategy) Instruction on how to perform behaviour (advise or agree on how to perform the behaviour (includes ‘Skills training’)) Information about antecedents (provide information about antecedents) Information about health consequences (provide information (e.g. written, verbal, visual) about health consequences of performing the behaviour) Salience of consequences (use methods specifically designed to emphasise the consequences of performing the behaviour with the aim of making them more memorable (goes beyond informing about consequences)) Information about social and environmental consequences (provide information (e.g. written, verbal, visual) about social and environmental consequences of performing the behaviour)
Cognitive and interpersonal skills (psychological capability)	HCPs perceived need for training to deliver behavioural support	*Training*	Guidelines Fiscal measures Regulation Legislation Service provision	Instruction on how to perform behaviour (advise or agree on how to perform the behaviour (includes ‘Skills training’)) Behavioural practice/rehearsal (prompt practice or rehearsal of the performance of the behaviour one or more times in a context or at a time when the performance may not be necessary, in order to increase habit and skill) Graded tasks (set easy-to-perform tasks, making them increasingly difficult, but achievable, until behaviour is performed)
	HCP training to communicate risk	*Training*	Guidelines Fiscal measures Regulation Legislation Service provision	Instruction on how to perform behaviour (advise or agree on how to perform the behaviour (includes ‘Skills training’)) Behavioural practice/rehearsal (prompt practice or rehearsal of the performance of the behaviour one or more times in a context or at a time when the performance may not be necessary, in order to increase habit and skill) Graded tasks (set easy-to-perform tasks, making them increasingly difficult, but achievable, until behaviour is performed)
Memory attention decision processes (psychological capability)	Behavioural intervention before pharmacological intervention	*Training* *Environmental restructuring* *Enablement*	Guidelines Fiscal measures Regulation Legislation Environmental/social planning (not training) Service provision (not environmental restructuring)	Prompts/cues (introduce or define environmental or social stimulus with the purpose of prompting or cueing the behaviour normally occurring at the time or place of performance) Conserving mental resources (advise on ways of minimising demands on mental resources to facilitate behaviour change)
Environmental context and resources (physical opportunity)	Limited time/ resources to deliver NHS Health Checks	*Training* *Restriction* *Environmental restructuring* *Enablement*	Guidelines Fiscal measures Regulation Legislation Environmental/ Social planning (not training or restriction) Service provision (not restriction or environmental restructuring)	Social support – practical (advise on, arrange, or provide practical help (e.g. from friends, relatives, colleagues, ‘buddies’ or staff) for performance of the behaviour) Prompts/cues (introduce or define environmental or social stimulus with the purpose of prompting or cueing the behaviour normally occurring at the time or place of performance) Remove aversive stimulus (advise or arrange for the removal of an aversive stimulus to facilitate behaviour change) Restructuring the physical environment (change, or advise to change the physical environment in order to facilitate performance of the wanted behaviour or create barriers to the unwanted behaviour (other than prompts/cues, rewards and punishments) Restructuring the social environment (change, or advise to change the social environment in order to facilitate performance of the wanted behaviour or create barriers to the unwanted behaviour (other than prompts/cues, rewards and punishments) Avoidance/reducing exposure to cues for the behaviour (advise on how to avoid exposure to specific social and contextual/physical cues for the behaviour, including changing daily or weekly routines) Adding objects to the environment (add objects to the environment in order to facilitate performance of the behaviour)
	Appropriate space to deliver NHS Health Checks	*Training* *Restriction* *Environmental restructuring* *Enablement*	Guidelines Fiscal measures Regulation Legislation Environmental/ Social planning (not training or restriction) Service provision (not restriction or environmental restructuring)	Social support – practical (advise on, arrange, or provide practical help (e.g. from friends, relatives, colleagues, ‘buddies’ or staff) for performance of the behaviour) Prompts/cues (introduce or define environmental or social stimulus with the purpose of prompting or cueing the behaviour normally occurring at the time or place of performance) Remove aversive stimulus (advise or arrange for the removal of an aversive stimulus to facilitate behaviour change) Restructuring the physical environment (change, or advise to change the physical environment in order to facilitate performance of the wanted behaviour or create barriers to the unwanted behaviour (other than prompts/cues, rewards and punishments) Restructuring the social environment (change, or advise to change the social environment in order to facilitate performance of the wanted behaviour or create barriers to the unwanted behaviour (other than prompts/cues, rewards and punishments) Avoidance/reducing exposure to cues for the behaviour (advise on how to avoid exposure to specific social and contextual/physical cues for the behaviour, including changing daily or weekly routines) Adding objects to the environment (add objects to the environment in order to facilitate performance of the behaviour)
	Computer systems supporting NHS Health Check delivery	*Training* *Restriction* *Environmental restructuring* *Enablement*	Guidelines Fiscal measures Regulation Legislation Environmental/ Social planning (not training or restriction) Service provision (not restriction or environmental restructuring)	Social support – practical (advise on, arrange, or provide practical help (e.g. from friends, relatives, colleagues, ‘buddies’ or staff) for performance of the behaviour) Prompts/cues (introduce or define environmental or social stimulus with the purpose of prompting or cueing the behaviour normally occurring at the time or place of performance) Remove aversive stimulus (advise or arrange for the removal of an aversive stimulus to facilitate behaviour change) Restructuring the physical environment (change, or advise to change the physical environment in order to facilitate performance of the wanted behaviour or create barriers to the unwanted behaviour (other than prompts/cues, rewards and punishments) Restructuring the social environment (change, or advise to change the social environment in order to facilitate performance of the wanted behaviour or create barriers to the unwanted behaviour (other than prompts/cues, rewards and punishments) Avoidance/reducing exposure to cues for the behaviour (advise on how to avoid exposure to specific social and contextual/physical cues for the behaviour, including changing daily or weekly routines) Adding objects to the environment (add objects to the environment in order to facilitate performance of the behaviour)
Social influences (social opportunity)	Taking account of patients’ social context	*Restriction* *Environmental restructuring* *Modelling* *Enablement*	Communication/ marketing (modelling only) Guidelines (not modelling) Fiscal measures (not modelling or restriction) Regulation (not modelling) Legislation (not modelling) Environmental/ Social planning (not modelling or restriction) Service provision (not restriction or environmental restructuring)	Social support – unspecified (advise on, arrange or provide social support (e.g. from friends, relatives, colleagues,’ buddies’ or staff) or non-contingent praise or reward for performance of the behaviour. It includes encouragement and counselling, but only when it is directed at the behaviour) Social support – practical (advise on, arrange, or provide practical help (e.g. from friends, relatives, colleagues, ‘buddies’ or staff) for performance of the behaviour) Social comparison (draw attention to others’ performance to allow comparison with the person’s own performance) Information about others’ approval (provide information about what other people think about the behaviour. The information clarifies whether others will like, approve or disapprove of what the person is doing or will do) Social reward (arrange verbal or non-verbal reward if and only if there has been eff ort and/or progress in performing the behaviour (includes ‘Positive reinforcement’))
Social/professional role and identity (reflective motivation)	Role clarity in delivering NHS Health Checks	*Education* *Persuasion* *Modelling*	Communication/ marketing Guidelines (not modelling) Regulation (not modelling) Legislation (not modelling) Service provision	Social support – unspecified (advise on, arrange or provide social support (e.g. from friends, relatives, colleagues,’ buddies’ or staff) or non-contingent praise or reward for performance of the behaviour. It includes encouragement and counselling, but only when it is directed at the behaviour)^b^ Social comparison (draw attention to others’ performance to allow comparison with the person’s own performance)^b^ Credible source (present verbal or visual communication from a credible source in favour of or against the behaviour)^b^ Identity associated with changed behaviour (advise the person to construct a new self-identity as someone who ‘used to engage with the unwanted behaviour’)^b^
	Diversification of pharmacy staff role	*Education* *Persuasion* *Modelling*	Communication/ marketing Guidelines (not modelling) Regulation (not modelling) Legislation (not modelling) Service provision	Social support – unspecified (advise on, arrange or provide social support (e.g. from friends, relatives, colleagues,’ buddies’ or staff) or non-contingent praise or reward for performance of the behaviour. It includes encouragement and counselling, but only when it is directed at the behaviour)^b^ Social comparison (draw attention to others’ performance to allow comparison with the person’s own performance)^b^ Credible source (present verbal or visual communication from a credible source in favour of or against the behaviour)^b^ Identity associated with changed behaviour (advise the person to construct a new self-identity as someone who ‘used to engage with the unwanted behaviour’)^b^
Beliefs about consequences (reflective motivation)	Belief that NHS Health Checks were beneficial in terms of preventive healthcare	*Education* *Persuasion* *Modelling*	Communication/ marketing Guidelines (not modelling) Regulation (not modelling) Legislation (not modelling) Service provision	Information about health consequences (provide information (e.g. written, verbal, visual) about health consequences of performing the behaviour) Salience of consequences (use methods specifically designed to emphasise the consequences of performing the behaviour with the aim of making them more memorable (goes beyond informing about consequences)) Information about social and environmental consequences (provide information (e.g. written, verbal, visual) about social and environmental consequences of performing the behaviour) Anticipated regret (induce or raise awareness of expectations of future regret about performance of the unwanted behaviour) Information about emotional consequences (provide information (e.g. written, verbal, visual) about emotional consequences of performing the behaviour) Pros and cons (advise the person to identify and compare reasons for wanting (pros) and not wanting to (cons) change the behaviour (includes ‘decisional balance’)) Comparative imagining of future outcomes (prompt or advise the imagining and comparing of future outcomes of changed versus unchanged behaviour) Material incentive - behaviour (inform that money, vouchers or other valued objects will be delivered if and only if there has been eff ort and/or progress in performing the behaviour (includes ‘Positive reinforcement’)) Incentive – outcome (inform that a reward will be delivered if and only if there has been eff ort and/or progress in achieving the behavioural outcome (includes ‘positive reinforcement’)) Reward - outcome (arrange for the delivery of a reward if and only if there has been eff ort and/or progress in achieving the behavioural outcome (includes ‘positive reinforcement’))
	Message framing	*Education* *Persuasion* *Modelling*	Communication/ marketing Guidelines (not modelling) Regulation (not modelling) Legislation (not modelling) Service provision	Information about health consequences (provide information (e.g. written, verbal, visual) about health consequences of performing the behaviour) Salience of consequences (use methods specifically designed to emphasise the consequences of performing the behaviour with the aim of making them more memorable (goes beyond informing about consequences)) Information about social and environmental consequences (provide information (e.g. written, verbal, visual) about social and environmental consequences of performing the behaviour) Anticipated regret (induce or raise awareness of expectations of future regret about performance of the unwanted behaviour) Information about emotional consequences (provide information (e.g. written, verbal, visual) about emotional consequences of performing the behaviour) Pros and cons (advise the person to identify and compare reasons for wanting (pros) and not wanting to (cons) change the behaviour (includes ‘decisional balance’)) Comparative imagining of future outcomes (prompt or advise the imagining and comparing of future outcomes of changed versus unchanged behaviour) Material incentive - behaviour (inform that money, vouchers or other valued objects will be delivered if and only if there has been eff ort and/or progress in performing the behaviour (includes ‘Positive reinforcement’)) Incentive – outcome (inform that a reward will be delivered if and only if there has been eff ort and/or progress in achieving the behavioural outcome (includes ‘positive reinforcement’)) Reward - outcome (arrange for the delivery of a reward if and only if there has been eff ort and/or progress in achieving the behavioural outcome (includes ‘positive reinforcement’))
Beliefs about capabilities (reflective motivation)	HCP confidence in discussing and initiating behaviour change	*Education* *Persuasion* *Modelling* *Enablement*	Communication/ marketing (not enablement) Guidelines (not modelling) Fiscal measures (enablement only) Regulation (not modelling) Legislation (not modelling) Environmental /social planning (enablement only) Service provision	Problem solving (analyse, or prompt the person to analyse, factors influencing the behaviour and generate or select strategies that include overcoming barriers and/or increasing facilitators (includes ‘relapse prevention’ and ‘coping planning’)) Instruction on how to perform behaviour (advise or agree on how to perform the behaviour (includes ‘Skills training’)) Demonstration of the behaviour (provide an observable sample of the performance of the behaviour, directly in person or indirectly e.g. via film, pictures, for the person to aspire to or imitate (includes ‘modelling’)) Behavioural practice/rehearsal (prompt practice or rehearsal of the performance of the behaviour one or more times in a context or at a time when the performance may not be necessary, in order to increase habit and skill) Graded tasks (set easy-to-perform tasks, making them increasingly difficult, but achievable, until behaviour is performed) Verbal persuasion about capability (tell the person that they can successfully perform the wanted behaviour, arguing against self-doubts and asserting that they can and will succeed) Focus on past success (advise to think about or list previous successes in performing the behaviour (or parts of it)) Self-talk (prompt positive self-talk (aloud or silently) before and during the behaviour)
Optimism (reflective motivation)	HCPs varyingly optimistic about patient behaviour change after NHS Health Checks	*Education* *Persuasion* *Modelling* *Enablement*	Communication/ marketing (not enablement) Guidelines (not modelling) Fiscal measures (enablement only) Regulation (not modelling) Legislation (not modelling) Environmental /social planning (enablement only) Service provision	Review outcome goal(s) (review outcome goal(s) jointly with the person and consider modifying goal(s) in light of achievement. This may lead to re-setting the same goal, a small change in that goal or setting a new goal instead of, or in addition to the first)^b^
HCP making referral to specialist service				
Environmental context and resources (physical opportunity)	Lack of funded services to refer patients to	*Training* *Restriction* *Environmental restructuring* *Enablement*	Guidelines Fiscal measures Regulation Legislation Environmental/ Social planning (not training or restriction) Service provision (not restriction or environmental restructuring)	Social support – practical (advise on, arrange, or provide practical help (e.g. from friends, relatives, colleagues, ‘buddies’ or staff) for performance of the behaviour) Prompts/cues (introduce or define environmental or social stimulus with the purpose of prompting or cueing the behaviour normally occurring at the time or place of performance) Remove aversive stimulus (advise or arrange for the removal of an aversive stimulus to facilitate behaviour change) Restructuring the physical environment (change, or advise to change the physical environment in order to facilitate performance of the wanted behaviour or create barriers to the unwanted behaviour (other than prompts/cues, rewards and punishments) Restructuring the social environment (change, or advise to change the social environment in order to facilitate performance of the wanted behaviour or create barriers to the unwanted behaviour (other than prompts/cues, rewards and punishments) Avoidance/reducing exposure to cues for the behaviour (advise on how to avoid exposure to specific social and contextual/physical cues for the behaviour, including changing daily or weekly routines) Adding objects to the environment (add objects to the environment in order to facilitate performance of the behaviour)
Patient attending referral				
Beliefs about consequences (reflective motivation)	Regular attendance is important	*Education* *Persuasion* *Modelling*	Communication/ marketing Guidelines (not modelling) Regulation (not modelling) Legislation (not modelling) Service provision	Information about health consequences (provide information (e.g. written, verbal, visual) about health consequences of performing the behaviour) Salience of consequences (use methods specifically designed to emphasise the consequences of performing the behaviour with the aim of making them more memorable (goes beyond informing about consequences)) Information about social and environmental consequences (provide information (e.g. written, verbal, visual) about social and environmental consequences of performing the behaviour) Anticipated regret (induce or raise awareness of expectations of future regret about performance of the unwanted behaviour) Information about emotional consequences (provide information (e.g. written, verbal, visual) about emotional consequences of performing the behaviour) Pros and cons (advise the person to identify and compare reasons for wanting (pros) and not wanting to (cons) change the behaviour (includes ‘decisional balance’)) Comparative imagining of future outcomes (prompt or advise the imagining and comparing of future outcomes of changed versus unchanged behaviour) Material incentive - behaviour (inform that money, vouchers or other valued objects will be delivered if and only if there has been eff ort and/or progress in performing the behaviour (includes ‘Positive reinforcement’)) Incentive – outcome (inform that a reward will be delivered if and only if there has been eff ort and/or progress in achieving the behavioural outcome (includes ‘positive reinforcement’)) Reward - outcome (arrange for the delivery of a reward if and only if there has been eff ort and/or progress in achieving the behavioural outcome (includes ‘positive reinforcement’))
Patient changing behaviour				
Knowledge (psychological capability)	Patient understanding of CVD risk and its implications after NHS Health Check	*Education*	Communication/ marketing Guidelines Regulation Legislation Service provision	Biofeedback (provide feedback about the body (e.g. physiological or biochemical state) using an external monitoring device as part of a behaviour change strategy) Instruction on how to perform behaviour (advise or agree on how to perform the behaviour (includes ‘Skills training’)) Information about antecedents (provide information about antecedents) Information about health consequences (provide information (e.g. written, verbal, visual) about health consequences of performing the behaviour) Salience of consequences (use methods specifically designed to emphasise the consequences of performing the behaviour with the aim of making them more memorable (goes beyond informing about consequences)) Information about social and environmental consequences (provide information (e.g. written, verbal, visual) about social and environmental consequences of performing the behaviour)
Environmental context and resources (physical opportunity)	Time and cost as a barrier to adherence	*Training* *Restriction* *Environmental restructuring* *Enablement*	Guidelines Fiscal measures Regulation Legislation Environmental/ Social planning (not training or restriction) Service provision (not restriction or environmental restructuring)	Social support – practical (advise on, arrange, or provide practical help (e.g. from friends, relatives, colleagues, ‘buddies’ or staff) for performance of the behaviour) Prompts/cues (introduce or define environmental or social stimulus with the purpose of prompting or cueing the behaviour normally occurring at the time or place of performance) Remove aversive stimulus (advise or arrange for the removal of an aversive stimulus to facilitate behaviour change) Restructuring the physical environment (change, or advise to change the physical environment in order to facilitate performance of the wanted behaviour or create barriers to the unwanted behaviour (other than prompts/cues, rewards and punishments) Restructuring the social environment (change, or advise to change the social environment in order to facilitate performance of the wanted behaviour or create barriers to the unwanted behaviour (other than prompts/cues, rewards and punishments) Avoidance/reducing exposure to cues for the behaviour (advise on how to avoid exposure to specific social and contextual/physical cues for the behaviour, including changing daily or weekly routines) Adding objects to the environment (add objects to the environment in order to facilitate performance of the behaviour)
	Adherence to behavioural support influenced by mode of communication of risk	*Training* *Restriction* *Environmental restructuring* *Enablement*	Guidelines Fiscal measures Regulation Legislation Environmental/ Social planning (not training or restriction) Service provision (not restriction or environmental restructuring)	Social support – practical (advise on, arrange, or provide practical help (e.g. from friends, relatives, colleagues, ‘buddies’ or staff) for performance of the behaviour) Prompts/cues (introduce or define environmental or social stimulus with the purpose of prompting or cueing the behaviour normally occurring at the time or place of performance) Remove aversive stimulus (advise or arrange for the removal of an aversive stimulus to facilitate behaviour change) Restructuring the physical environment (change, or advise to change the physical environment in order to facilitate performance of the wanted behaviour or create barriers to the unwanted behaviour (other than prompts/cues, rewards and punishments) Restructuring the social environment (change, or advise to change the social environment in order to facilitate performance of the wanted behaviour or create barriers to the unwanted behaviour (other than prompts/cues, rewards and punishments) Avoidance/reducing exposure to cues for the behaviour (advise on how to avoid exposure to specific social and contextual/physical cues for the behaviour, including changing daily or weekly routines) Adding objects to the environment (add objects to the environment in order to facilitate performance of the behaviour)
Social influences (social opportunity)	Support from others to change	*Restriction* *Environmental restructuring* *Modelling* *Enablement*	Communication/ marketing (modelling only) Guidelines (not modelling) Fiscal measures (not modelling or restriction) Regulation (not modelling) Legislation (not modelling) Environmental/ Social planning (not modelling or restriction) Service provision (not restriction or environmental restructuring)	Social support – unspecified (advise on, arrange or provide social support (e.g. from friends, relatives, colleagues,’ buddies’ or staff) or non-contingent praise or reward for performance of the behaviour. It includes encouragement and counselling, but only when it is directed at the behaviour) Social support – practical (advise on, arrange, or provide practical help (e.g. from friends, relatives, colleagues, ‘buddies’ or staff) for performance of the behaviour) Social comparison (draw attention to others’ performance to allow comparison with the person’s own performance) Information about others’ approval (provide information about what other people think about the behaviour. The information clarifies whether others will like, approve or disapprove of what the person is doing or will do) Social reward (arrange verbal or non-verbal reward if and only if there has been eff ort and/or progress in performing the behaviour (includes ‘Positive reinforcement’))
Social/professional role and identity (reflective motivation)	Patient engagement is influenced by HCP role	*Education* *Persuasion* *Modelling*	Communication/ marketing Guidelines (not modelling) Regulation (not modelling) Legislation (not modelling) Service provision	Social support – unspecified (advise on, arrange or provide social support (e.g. from friends, relatives, colleagues,’ buddies’ or staff) or non-contingent praise or reward for performance of the behaviour. It includes encouragement and counselling, but only when it is directed at the behaviour)^b^ Social comparison (draw attention to others’ performance to allow comparison with the person’s own performance)^b^ Credible source (present verbal or visual communication from a credible source in favour of or against the behaviour)^b^ Identity associated with changed behaviour (advise the person to construct a new self-identity as someone who ‘used to engage with the unwanted behaviour’)^b^
Beliefs about capabilities (reflective motivation)	Changes perceived to be achievable	*Education* *Persuasion* *Modelling* *Enablement*	Communication/ marketing (not enablement) Guidelines (not modelling) Fiscal measures (enablement only) Regulation (not modelling) Legislation (not modelling) Environmental /social planning (enablement only) Service provision	Problem solving (analyse, or prompt the person to analyse, factors influencing the behaviour and generate or select strategies that include overcoming barriers and/or increasing facilitators (includes ‘relapse prevention’ and ‘coping planning’)) Instruction on how to perform behaviour (advise or agree on how to perform the behaviour (includes ‘Skills training’)) Demonstration of the behaviour (provide an observable sample of the performance of the behaviour, directly in person or indirectly e.g. via film, pictures, for the person to aspire to or imitate (includes ‘modelling’)) Behavioural practice/rehearsal (prompt practice or rehearsal of the performance of the behaviour one or more times in a context or at a time when the performance may not be necessary, in order to increase habit and skill) Graded tasks (set easy-to-perform tasks, making them increasingly difficult, but achievable, until behaviour is performed) Verbal persuasion about capability (tell the person that they can successfully perform the wanted behaviour, arguing against self-doubts and asserting that they can and will succeed) Focus on past success (advise to think about or list previous successes in performing the behaviour (or parts of it)) Self-talk (prompt positive self-talk (aloud or silently) before and during the behaviour)
Beliefs about consequences (reflective motivation)	Contradictory guidelines	*Education* *Persuasion* *Modelling*	Communication/ marketing Guidelines (not modelling) Regulation (not modelling) Legislation (not modelling) Service provision	Information about health consequences (provide information (e.g. written, verbal, visual) about health consequences of performing the behaviour) Salience of consequences (use methods specifically designed to emphasise the consequences of performing the behaviour with the aim of making them more memorable (goes beyond informing about consequences)) Information about social and environmental consequences (provide information (e.g. written, verbal, visual) about social and environmental consequences of performing the behaviour) Anticipated regret (induce or raise awareness of expectations of future regret about performance of the unwanted behaviour) Information about emotional consequences (provide information (e.g. written, verbal, visual) about emotional consequences of performing the behaviour) Pros and cons (advise the person to identify and compare reasons for wanting (pros) and not wanting to (cons) change the behaviour (includes ‘decisional balance’)) Comparative imagining of future outcomes (prompt or advise the imagining and comparing of future outcomes of changed versus unchanged behaviour) Material incentive - behaviour (inform that money, vouchers or other valued objects will be delivered if and only if there has been eff ort and/or progress in performing the behaviour (includes ‘Positive reinforcement’)) Incentive – outcome (inform that a reward will be delivered if and only if there has been eff ort and/or progress in achieving the behavioural outcome (includes ‘positive reinforcement’)) Reward - outcome (arrange for the delivery of a reward if and only if there has been eff ort and/or progress in achieving the behavioural outcome (includes ‘positive reinforcement’))
	Perceptions of what constitutes healthy behaviour	*Education* *Persuasion* *Modelling*	Communication/ marketing Guidelines (not modelling) Regulation (not modelling) Legislation (not modelling) Service provision	Information about health consequences (provide information (e.g. written, verbal, visual) about health consequences of performing the behaviour) Salience of consequences (use methods specifically designed to emphasise the consequences of performing the behaviour with the aim of making them more memorable (goes beyond informing about consequences)) Information about social and environmental consequences (provide information (e.g. written, verbal, visual) about social and environmental consequences of performing the behaviour) Anticipated regret (induce or raise awareness of expectations of future regret about performance of the unwanted behaviour) Information about emotional consequences (provide information (e.g. written, verbal, visual) about emotional consequences of performing the behaviour) Pros and cons (advise the person to identify and compare reasons for wanting (pros) and not wanting to (cons) change the behaviour (includes ‘decisional balance’)) Comparative imagining of future outcomes (prompt or advise the imagining and comparing of future outcomes of changed versus unchanged behaviour) Material incentive - behaviour (inform that money, vouchers or other valued objects will be delivered if and only if there has been eff ort and/or progress in performing the behaviour (includes ‘Positive reinforcement’)) Incentive – outcome (inform that a reward will be delivered if and only if there has been eff ort and/or progress in achieving the behavioural outcome (includes ‘positive reinforcement’)) Reward - outcome (arrange for the delivery of a reward if and only if there has been eff ort and/or progress in achieving the behavioural outcome (includes ‘positive reinforcement’))
Intentions (reflective motivation)	NHS Health Checks as a ‘wake-up call’	*Education* *Persuasion* *Incentivisation* *Coercion* *Modelling*	Communication/ marketing Guidelines (not modelling) Fiscal measures (incentivisation and coercion only) Regulation (not modelling) Legislation (not modelling) Service provision	Goal setting - behaviour (set or agree a goal defined in terms of the behaviour to be achieved) Information about health consequences (provide information (e.g. written, verbal, visual) about health consequences of performing the behaviour) Incentive – outcome (inform that a reward will be delivered if and only if there has been eff ort and/or progress in achieving the behavioural outcome (includes ‘positive reinforcement’))
Optimism (reflective motivation)	Fatalistic views about disease	*Education* *Persuasion* *Modelling* *Enablement*	Communication/ marketing (not enablement) Guidelines (not modelling) Fiscal measures (enablement only) Regulation (not modelling) Legislation (not modelling) Environmental /social planning (enablement only) Service provision	Review outcome goal(s) (review outcome goal(s) jointly with the person and consider modifying goal(s) in light of achievement. This may lead to re-setting the same goal, a small change in that goal or setting a new goal instead of, or in addition to the f
Patient attending repeat NHS Health Check				
Intentions (reflective motivation)	Likelihood of attending future NHS Health Checks	*Education* *Persuasion* *Incentivisation* *Coercion* *Modelling*	Communication/ marketing Guidelines (not modelling) Fiscal measures (incentivisation and coercion only) Regulation (not modelling) Legislation (not modelling) Service provision	Goal setting - behaviour (set or agree a goal defined in terms of the behaviour to be achieved) Information about health consequences (provide information (e.g. written, verbal, visual) about health consequences of performing the behaviour) Incentive – outcome (inform that a reward will be delivered if and only if there has been eff ort and/or progress in achieving the behavioural outcome (includes ‘positive reinforcement’))
HCP recording NHS Health Check data				
Environmental context and resources (physical opportunity)	Accuracy of recording is compromised by multiple methods of invitation	*Training* *Restriction* *Environmental restructuring* *Enablement*	Guidelines Fiscal measures Regulation Legislation Environmental/ Social planning (not training or restriction) Service provision (not restriction or environmental restructuring)	Social support – practical (advise on, arrange, or provide practical help (e.g. from friends, relatives, colleagues, ‘buddies’ or staff) for performance of the behaviour) Prompts/cues (introduce or define environmental or social stimulus with the purpose of prompting or cueing the behaviour normally occurring at the time or place of performance) Remove aversive stimulus (advise or arrange for the removal of an aversive stimulus to facilitate behaviour change) Restructuring the physical environment (change, or advise to change the physical environment in order to facilitate performance of the wanted behaviour or create barriers to the unwanted behaviour (other than prompts/cues, rewards and punishments) Restructuring the social environment (change, or advise to change the social environment in order to facilitate performance of the wanted behaviour or create barriers to the unwanted behaviour (other than prompts/cues, rewards and punishments) Avoidance/reducing exposure to cues for the behaviour (advise on how to avoid exposure to specific social and contextual/physical cues for the behaviour, including changing daily or weekly routines) Adding objects to the environment (add objects to the environment in order to facilitate performance of the behaviour)

^a^Based on the matrix in Additional file 3 linking COM-B to potentially relevant intervention functions, we used links suggested in Michie, Atkins & West (2014) for which intervention functions might be relevant to TDF domains. This allowed for more specific recommendations

^b^No conclusive links were identified between BCTs and TDF domain so BCTs for which there was inconclusive evidence of links are presented

**Table 7:** BCTs to target key barriers to and facilitators of NHS Health Check behaviours

Behaviour	Key influence (barrier/facilitator coded by COM-B and TDF)	Examples of BCT delivery
HCPs delivering NHS Health Check	Behavioural intervention before pharmacological intervention	Prompts/cues: A stimulus to prompt or cue the behaviour, e.g. screen pop-up reminding HCP to offer behavioural support first.
	COM-B: Psychological capability	Conserving mental resources: Minimising demands on mental resources. This BCT may not be relevant in this context.
	TDF: Memory, attention decision processes	
	HCP familiarity with relevant evidence-based guidelines	Biofeedback (definition: provide feedback about the body using an external monitoring device): Pedometer linked to app.
	COM-B: Psychological capability	Instruction on how to perform behaviour: provide HCPs with electronic summaries of relevant guidelines.
	TDF: Knowledge	Information about antecedents (definition: provide information about social and environmental situations and events, emotions, cognitions that predict performance of the behaviour): Text on website or app suggesting HCPs be mindful of the situations, such as busy clinics, where guideline content may be over-looked.
		Information about health consequences: Electronic content explaining to HCPs that adhering to EBGs can improve patient health outcomes or presenting data (if available) on improvements in health outcomes as a result of adhering to EBGs.
		Salience of consequences (definition: use methods specifically designed to emphasise the consequences of performing the behaviour with the aim of making them more memorable such as by using imagery or metaphor): Include a picture of relevant guidelines with text underneath ‘if you know the contents of this’ and picture of person having heart attack with text underneath ‘then you are more likely to prevent this.’
		Information about social and environmental consequences: Electronic content providing data on costs saved to workforce by adhering to EBGs to prevent heart attacks.
	HCP confidence in discussing and initiating behaviour change	Instruction on how to perform behaviour: Electronic summaries of strategies to bring about behaviour change with examples (summary of strategy = setting a goal, example = agree with the patient they will walk for 30 mins three times a week).
	COM-B: Reflective motivation	Demonstration of the behaviour: Video of consultation or role play.
	TDF: Beliefs about capabilities	Behavioural practice/rehearsal: Text on website/ app (ideally near video demonstrating delivery of behaviour change strategies) encouraging HCPs to participate in role play with patient / colleague / family / friend.
		Focus on past success: Text on website/ app encouraging HCP to think of examples where they have successfully supported a patient to change their behaviour.
	Framing CVD risk messages	Information about health consequences: Provide data or text summary on website/app linking improved patient outcomes to understanding CVD.
	COM-B: Reflective motivation	Salience of consequences: This BCT may not be relevant in this context.
	TDF: Beliefs about consequences	Information about social and environmental consequences: On website/app include text explain to HCPs that the majority of HCPs approve of appropriate risk framing messages.
		Anticipated regret: On website/app include text asking HCPs to imagine how they would feel if patients did not act to reduce their CVD risk based on inaccurately framed messages.
		Information about emotional consequences: Quotes or videos from patient talking about how HCPs inaccurately conveying CVD risk made them overly anxious.
		Pros and cons: On website/app include text asking HCPs to list the advantages and disadvantages of over or underplaying CVD risk (or provide these lists and ask HCPs to think of more).
		Comparative imagining of future outcomes: On website/app include text asking HCPs to compare what would happen if they appropriately framed the communication of risk compared with what they currently do.
		Material incentive (behaviour): Inform HCPs that a financial payment will be made for each Health Check where risk is framed appropriately, e.g. according to an agreed protocol.
		Incentive (outcome): Inform HCPs that a financial payment will be made if a patient changes their behaviour as a result of an appropriately framed CVD risk message.
		Reward (outcome): Arrange for a financial payment to HCPs if a patient changes their behaviour as a result of the HCP appropriately framing CVD risk.
	Time/resources to deliver Health Checks	Social support (practical): On website/app include text suggesting HCPs arrange for colleagues to take on some of the HCP’s duties to free up their time to deliver Health Checks.
	COM-B: Physical opportunity	Prompts/cues: This BCT may not be relevant in this context.
	TDF: Environmental context and resources	Remove aversive stimulus: This BCT may not be relevant in this context.
		Restructuring the physical environment: On website/app include text suggesting HCPs ask service managers to restructure clinics to offer longer appointment times for Health Checks.
		Restructuring the social environment: This BCT may not be relevant in this context.
		Avoidance/reducing exposure to cues for the behaviour: This BCT may not be relevant in this context.
		Adding objects to the environment: Provide HCPs with electronic schedules to guide timely delivery of Health Checks, e.g. ‘spend no more than 2 min on…... ‘
Patient behaviour change after NHS Health Check	Knowledge of CVD risk and implication	Biofeedback: This BCT may not be relevant in this context.
	COM-B: Psychological capability	Instruction on how to perform behaviour: Provide patients with electronically available summaries of information necessary to interpret their risk scores.
	TDF: Knowledge	Information about antecedents: This BCT may not be relevant in this context.
		Information about health consequences: Provide patients with electronic information on the implications of their risk score, e.g. ‘x out of x people with a QRisk score of 11 are likely to experience stroke/other cardiovascular event.’ (risk score calculator?)
		Salience of consequences: On website/app include text include diagrams of arteries of individuals with low to high cholesterol.
		Information about social and environmental consequences: On website/app include text about how their risk might impact on their friendships, family or work.
	The NHS Health Check as a Wake-up call for change	Goal setting (behaviour): Include space on an app/website to set a goal, e.g. get off the bus two stops early and walk the rest of the way to work)
	COM-B: Reflective motivation	Information about health consequences: Quotes and/or videos from patients talking about the health benefits of the changes they have made.
	TDF: Intentions	Incentive (outcome): On website/app include text informing patients that a financial payment will be made only if their QRisk score changes as a result of dietary/physical activity changes.
	Support from others to change	Social support (unspecified): On website/app include text suggesting patients to ask a colleague to agree in advance to take the ‘healthy option’ lunch in the work canteen.
	COM-B: Social opportunity	Social support (practical): On website/app include text encouraging patients to ask friends for help with arrangements to accommodate their health goals, e.g. asking a friend to look after patient’s children while they go swimming.
	TDF: Social influences	Social comparison: Quotes and/or videos from patients talking about how they have increased their physical activity.
		Information about others’ approval: Include quotes and/or videos from other patients talking about how supportive family/friends have been of the changes they have made. If functionality allows - invite family/friends to post encouraging messages (video or text).
		Social reward: If an app/website is tracking patients progress, sending a message of congratulations for any changes made.
	The extent to which patients believe change is achievable	Problem solving: In app/website provide a list of common barriers to change and some possible solutions and encourage patient to generate their own, e.g. lack of motivation could be addressed with going to the gym with a buddy.
	COM-B: Reflective motivation	Instruction on how to perform behaviour: Provide patients with a list of lines to initiate conversation with a partner about changing diet and tips on how to avoid, e.g. partner being resistance to change.
	TDF: Beliefs about capabilities	Demonstration of the behaviour: Video of patient talking to their partner about changing diet and negotiating possible barriers.
		Behavioural practice/rehearsal: Text on website/ app (ideally near video demonstrating talking about change with a partner) encouraging patients to practice with a friend.
		Graded tasks: Include text/video conveying that change is more likely if you build on small successes and suggesting how this might be done, e.g. walk for 100 yards a day for the first week, then half a mile a day after they have successfully achieved 100 yards, then two miles a day after they have successfully achieved one mile.
		Verbal persuasion about capability: Include text/video of a patient who has successful changed their behaviour telling the patient they can also successfully increase their physical activity.
		Focus on past success: Include text/video encouraging the patient to think of examples where they have successfully changed their behaviour.
		Self-talk: Include text/video prompting the patient to tell themselves that a walk will be energising.

## Discussion

We identified nine behaviours related to NHS Health Checks and barriers to and facilitators of eight of these behaviours): i) HCPs inviting patients to attend NHS Health Checks - It can be time consuming identifying eligible patients; ii) patients attending NHS Health Checks - Family history of illness, the need to reduce anxiety and be reassured may increase attendance. Since patients need to perceive the NHS Health Check as relevant to them, this may be facilitated by patients understanding the purpose of NHS Health Checks as an opportunity to be proactive about CVD.; iii) HCPs delivering NHS Health Checks - HCPs perceive the need for more training (risk communication and behaviour change) and time and appropriate space to deliver NHS Health Checks. HCPs disagree on the extent to which NHS Health Checks were beneficial to patients and whether offering behavioural before pharmacological intervention was appropriate. Taking account of patients’ social context and appropriate message framing are perceived as important but do not always happen. HCPs had varying levels of confidence to deliver behavioural support.; iv) HCPs refer patients to a specialist service - Lack of availability of relevant services hinder onward referral; v) patients attending specialist referral - Patients believe it is important to attend appointments regularly; vi) patients changing CVD risk-related behaviours - NHS Health Checks can serve as a ‘wake-up call’ to change. However, patients vary in their understanding of CVD risk following NHS Health Check and some are not aware of the behaviours which can influence CVD risk. The influence of family and friends in supporting change is perceived as important and HCP role can influence change differentially. Fatalistic beliefs in health can hinder change as can contradictory guidelines. Patients welcome small, incremental changes to behaviour.; vii) patients attending repeat NHS Health Check - Most patients intend to attend a repeat NHS Health Check; viii) HCPs recording NHS Health Check data - Recording relevant data can be hindered where multiple invitation methods are used; ix) service managers and/or commissioners synthesising and disseminating NHS Health Check data (no barriers or facilitators were identified in relation to this behaviour).

This is the first study, to our knowledge, to apply theoretical models to studies focussing solely on influences on NHS Health Check behaviours and to make theory-based recommendations for intervention design. Shaw et al. 2016 [54] used the TDF to understand NHS Health Check-related behaviours in a review but included UK and non-UK CVD risk reduction programmes.

Implications for service commissioners and providers are to audit current provision against the behaviours, barriers and facilitators identified in this study. This would provide foundations in ensuring the programme remains fit for purpose for the next 10 years and beyond. Implications for commissioners are that they should maximise opportunities to ensure that the purpose of NHS Health Checks as a prevention programme is clear to eligible patients in promotional materials, provide digital records structured to easily identify eligible patients and record programme data. Implications for HCPs providing NHS Health Checks are that they should offer flexible appointment times where possible, invest in training for frontline staff to provide behavioural support for patients and ensure that the benefits of NHS Health Checks are conveyed to staff.

It should be noted that the review is only as comprehensive as the studies it includes, and these have not all taken a comprehensive approach to investigating barriers and facilitators, i.e. they may present a partial picture of influences on behaviours. Our review findings may therefore reflect an absence of evidence (i.e. a TDF domain reported as not relevant to a behaviour may be because it was not investigated) rather than an evidence of absence (a TDF domain was investigated and found not to be relevant to a behaviour). It should also be noted that the recommendations flowing from our findings reflect the types of interventons that we would expect on theoretical grounds to be effective, and this is not a review of effectiveness of interventions; that would also be a useful study.

Such a study could investigate the extent to which interventions to promote NHS Health Check behaviours target the barriers and facilitators identified in this review. This work would identify any missed opportunities for intervention and inform intervention design and refinement. Interventions (both nationally and locally implemented) to promote NHS Health Check behaviours could be described in terms of function and channels through which interventions are delivered using the frameworks of BCW and BCTTv1. This would include describing interventons in terms of their service provision such as pharmacy and apps and local outreach; communication and marketing in NHS Choices and NHS Health Check invitation letters; regulation and legislation for the NHS Health Check programme; and guidelines for delivery of the NHS Health Check appointment.

A further study that would add to this evidence is a consensus study of experts in the various relevant areas to ascertain the extent to which they agree with the findings from the literature, to identiy gaps and to elaborate with likely ranges of effective application of each proposed intervention.

The NHS Health Check programme has seen 6.7 million people aged 40 to 74 benefit from a check over the past 5 years. This study identifies key influencers of behaviour which impact on the success of the programme based on a systematic review that was strengthened by coding of extracted data into behavioural frameworks and formation of a detailed underpinning user journey and Theory of Change for the NHS Health Check programme. Based on this rigorous analysis, it makes specific, evidence-informed recommendations for commissioners, which should be considered as part of the forthcoming NHS Health Check review in order to ensure the programme is implemented as effectively as possible for the next 10 years and beyond [55].

## Conclusion

To the authors’ knowledge, this is the first attempt to apply the behavioural science frameworks, BCW, TDF and BCTTv1 to understand influences on NHS Health Check behaviours and systematically identify likely effective interventions and component techniques that could be either incorporated into existing interventions or form the basis of designing new interventions.

## Supplementary information

**Additional file 1:.** COM-B and TDF labels.

**Additional file 2:.** BCW labels.

**Additional file 3:.** BCW matrices.

**Additional file 4:.** BCT TDF matrix.

**Additional file 5:.** Search terms.

**Additional file 6:.** MMAT study quality.

**Additional file 7:.** APEASE criteria.

## Data Availability

The datasets used and/or analysed during the current study are available from the corresponding author on reasonable request.

## Abbreviations

BCT: Behaviour change technique
BCTTv1: Behaviour Change Techniques Taxonomy v1
BCW: Behaviour Change Wheel
COM-B: Capability Opportunity Motivation – Behaviour
CVD: Cardiovascular disease
HCP: Healthcare professional
MMAT: Mixed Methods Appraisal Tool
NHS: National Health Service
PHE: Public Health England
TDF: Theoretical Domains Framework

## Footnotes

1 British Heart Foundation (www.bhf.org.uk/-/media/files/research/heart-statistics/bhf-cvd-statistics%2D%2D-uk-factsheet.pdf)
