# Supplementary material for: Influences on NHS Health Check behaviours: a systematic review
Source: BMC Public Health. 2020 Sep 17;20:1359. doi: 10.1186/s12889-020-09365-2 (PMC7495879; doi:10.1186/s12889-020-09365-2)
Supplement: Supplementary file 1 — Additional file 1:. COM-B and TDF labels. [file 12889_2020_9365_MOESM1_ESM.docx]

**Additional file 1: Labels, definitions and examples of COM-B and Theoretical Domains Framework**

**COM-B model**

| **COM-B model component**  **Definition** | ***Example*** |
| --- | --- |
| Physical capability  Physical skill, strength or stamina | *Having the skill to take a blood sample* |
| Psychological capability  Knowledge or psychological skills, strength or stamina to engage in the necessary mental processes | *Understanding the impact of CO^2^ on the environment* |
| Physical opportunity  Opportunity afforded by the environment involving time, resources, locations, cues, physical ‘affordance’ | *Being able to go running because one owns appropriate shoes* |
| Social opportunity  Opportunity afforded by interpersonal influences, social cues and cultural norms that influence the way that we think about things, e.g. the words and concepts that make up our language | *Being able to smoke in the house of someone who smokes but not in the middle of a boardroom meeting* |
| Reflective motivation  Reflective processes involving plans (self-conscious intentions) and evaluations (beliefs about what is good and bad) | *Intending to stop smoking* |
| Automatic motivation  Automatic processes involving emotional reactions, desires (wants and needs), impulses, inhibitions, drive states and reflex responses | *Feeling anticipated pleasure at the prospect of eating a piece of chocolate cake* |

**Theoretical domains framework**

| **Domain**  **Definition** | **Theoretical constructs represented within each domain** | ***Interview questions**** | |
| --- | --- | --- | --- |
| Knowledge  An awareness of the existence of something | Knowledge (including knowledge of condition /scientific rationale); procedural knowledge; knowledge of task environment | *Do you know about x?* | |
| Skills  An ability or proficiency acquired through practice | Skills; skills development; competence; ability; interpersonal skills; practice; skill assessment | *Do you know how to do x?* | |
| Memory, attention and decision Processes  The ability to retain information, focus selectively on aspects of the environment and choose between two or more alternatives | Memory; attention; attention control; decision making; cognitive overload / tiredness | *Is x something you usually do?* | |
| Behavioural regulation  Anything aimed at managing or changing objectively observed or measured actions | Self-monitoring; breaking habit; action planning | *Do you have systems that you could use for monitoring whether or not you have carried x?* | |
| Social/professional role and identity  A coherent set of behaviours and displayed personal qualities of an individual in a social or work setting | Professional identity; professional role; social identity; identity; professional boundaries; professional confidence; group identity; leadership; organisational commitment | *Is doing x compatible or in conflict with professional standards/identity?* | |
| Beliefs about capabilities  Acceptance of the truth, reality, or validity about an ability, talent, or facility that a person can put to constructive use | Self-confidence; perceived competence; self-efficacy; perceived behavioural control; beliefs; self-esteem; empowerment; professional confidence | *How difficult or easy is it for you to do x?* | |
| Optimism  The confidence that things will happen for the best or that desired goals will be attained | Optimism; pessimism; unrealistic optimism; identity | *How confident are you that the problem of implementing x will be solved?* | |
| Beliefs about consequences  Acceptance of the truth, reality, or validity about outcomes of a behaviour in a given situation) | Beliefs; outcome expectancies; characteristics of outcome expectancies; anticipated regret; consequents | *What do you think will happen if you do x?* | |
| Intentions  A conscious decision to perform a behaviour or a resolve to act in a certain way | Stability of intentions; stages of change model; transtheoretical model and stages of change | *Have they made a decision to do x?* | |
| Goals  Mental representations of outcomes or end states that an individual wants to achieve | Goals (distal / proximal) ; goal priority; goal / target setting; goals (autonomous / controlled); action planning; implementation intention | *How much do they want to do x?* | |
| Reinforcement  Increasing the probability of a response by arranging a dependent relationship, or contingency, between the response and a given stimulus | Rewards (proximal / distal, valued / not valued, probable / improbable); incentives; punishment; consequents; reinforcement; contingencies; sanctions | *Are there incentives to do x?* | |
| Emotion  A complex reaction pattern, involving experiential, behavioural, and physiological elements, by which the individual attempts to deal with a personally significant matter or event | Fear; anxiety; affect; stress; depression; positive / negative affect; burn-out | *Does doing x evoke an emotional response?* | |
| Environmental context and resources  Any circumstance of a person's situation or environment that discourages or encourages the development of skills and abilities, independence, social competence, and adaptive behaviour | Environmental stressors ; resources / material resources ; organisational culture /climate ; salient events / critical incidents; person x environment interaction; barriers and facilitators | *To what extent do physical or resource factors facilitate or hinder x?* | |
| Social influences  Those interpersonal processes that can cause individuals to change their thoughts, feelings, or behaviours | Social pressure; social norms; group conformity; social comparisons; group norms; social support; power; intergroup conflict; alienation; group identity; modelling | *To what extent do social influences facilitate or hinder x?* | |
| * Summarised from Michie et al. (2005) | | |  |
