# Supplementary material for: Influences on NHS Health Check behaviours: a systematic review
Source: BMC Public Health. 2020 Sep 17;20:1359. doi: 10.1186/s12889-020-09365-2 (PMC7495879; doi:10.1186/s12889-020-09365-2)
Supplement: Supplementary file 2 — Additional file 2:. BCW labels. [file 12889_2020_9365_MOESM2_ESM.docx]

# Additional file 2: Behaviour Change Wheel labels, definitions and examples

**Intervention types**

| **Intervention type** | **Definition** | ***Example*** |
| --- | --- | --- |
| Education | Increasing knowledge or understanding | *Providing information to promote healthy eating* |
| Persuasion | Using communication to induce positive or negative feelings or stimulate action | *Using imagery to motivate increases in physical activity* |
| Incentivisation | Creating an expectation of reward | *Using prize draws to induce attempts to stop smoking* |
| Coercion | Creating an expectation of punishment or cost | *Raising the financial cost to reduce excessive alcohol consumption* |
| Training | Imparting skills | *Advanced driver training to increase safe driving* |
| Restriction | Using rules to reduce the opportunity to engage in the target behaviour (or to increase the target behaviour by reducing the opportunity to engage in competing behaviours) | *Prohibiting sales of solvents to people under 18 to reduce use for intoxication* |
| Environmental restructuring | Changing the physical or social context | *Providing on-screen prompts for GPs to ask about smoking behaviour* |
| Modelling | Providing an example for people to aspire to or imitate | *Using TV drama scenes involving safe-sex practices to increase condom use* |
| Enablement | Increasing means/reducing barriers to increase capability (beyond education and training) or opportunity (beyond environmental restructuring) | *Behavioural support for smoking cessation, medication for cognitive deficits, surgery to reduce obesity, prostheses to promote physical activity* |

**Policy options**

| **Policy options** | **Definition** | **Example** |
| --- | --- | --- |
| Communication/ marketing | Using print, electronic, telephonic or broadcast media | *Conducting mass media campaigns* |
| Guidelines | Creating documents that recommend or mandate practice. This includes all changes to service provision | *Producing and disseminating treatment protocols* |
| Fiscal measures | Using the tax system to reduce or increase the financial cost | *Increasing duty or increasing anti-smuggling activities* |
| Regulation | Establishing rules or principles of behaviour or practice | *Establishing voluntary agreements on advertising* |
| Legislation | Making or changing laws | *Prohibiting sale or use* |
| Environmental/social planning | Designing and/or controlling the physical or social environment | *Using town planning* |
| Service provision | Delivering a service | *Establishing support services in workplaces, communities etc.* |
