# Supplementary material for: Influences on NHS Health Check behaviours: a systematic review
Source: BMC Public Health. 2020 Sep 17;20:1359. doi: 10.1186/s12889-020-09365-2 (PMC7495879; doi:10.1186/s12889-020-09365-2)
Supplement: Supplementary file 3 — Additional file 3:. BCW matrices. [file 12889_2020_9365_MOESM3_ESM.docx]

**Additional file 3: Suggested links between COM-B/TDF, intervention types and policy options**

**Intervention types to consider based on COM-B coded influence on behaviour (Michie et al. 2011)**

| **COM-B (TDF)** | **Intervention types** | | | | | | | | |
| --- | --- | --- | --- | --- | --- | --- | --- | --- | --- |
|  | Education | Persuasion | Incentivisation | Coercion | Training | Restriction | Environmental restructuring | Modelling | Enablement |
| Physical Capability |  |  |  |  |  |  |  |  |  |
| Psychological Capability |  |  |  |  |  |  |  |  |  |
| Physical Opportunity |  |  |  |  |  |  |  |  |  |
| Social Opportunity |  |  |  |  |  |  |  |  |  |
| Automatic Motivation |  |  |  |  |  |  |  |  |  |
| Reflective Motivation |  |  |  |  |  |  |  |  |  |

**Policy options to consider based on Intervention types (Michie et al. 2011)**

| **Intervention types** | **Policy options** | | | | | | |
| --- | --- | --- | --- | --- | --- | --- | --- |
|  | Communication/ marketing | Guidelines | Fiscal measures | Regulation | Legislation | Environmental/ Social planning | Service provision |
| Education |  |  |  |  |  |  |  |
| Persuasion |  |  |  |  |  |  |  |
| Incentivisation |  |  |  |  |  |  |  |
| Coercion |  |  |  |  |  |  |  |
| Training |  |  |  |  |  |  |  |
| Restriction |  |  |  |  |  |  |  |
| Environmental restructuring |  |  |  |  |  |  |  |
| Modelling |  |  |  |  |  |  |  |
| Enablement |  |  |  |  |  |  |  |

**Intervention types to consider based on TDF coded influence on behaviour (Michie et al. 2014)**

| **COM-B** | **TDF** | **Intervention types** |
| --- | --- | --- |
| Physical capability | Physical skills | Training |
| Psychological capability | Knowledge | Education |
|  | Cognitive and interpersonal skills | Training |
|  | Memory, attention and decision processes | Training Environmental restructuring Enablement |
|  | Behavioural regulation | Education Training Modelling Enablement |
| Reflective motivation | Social/professional role and identity | Education Persuasion Modelling |
|  | Beliefs about capabilities | Education Persuasion Modelling Enablement |
|  | Optimism | Education Persuasion Modelling Enablement |
|  | Beliefs about consequences | Education Persuasion Modelling |
|  | Intentions | Education Persuasion Incentivisation Coercion Modelling |
|  | Goals | Education Persuasion Incentivisation Coercion Modelling  Enablement |
| Automatic motivation | Reinforcement | Training  Incentivisation Coercion Environmental restructuring |
|  | Emotion | Persuasion Incentivisation Coercion Modelling Enablement |
| Physical opportunity | Environmental context and resources | Training  Restriction Environmental restructuring Enablement |
| Social opportunity | Social influences | Restriction Environmental restructuring Modelling Enablement |
