# Supplementary material for: Influences on NHS Health Check behaviours: a systematic review
Source: BMC Public Health. 2020 Sep 17;20:1359. doi: 10.1186/s12889-020-09365-2 (PMC7495879; doi:10.1186/s12889-020-09365-2)
Supplement: Supplementary file 4 — Additional file 4:. BCT TDF matrix. [file 12889_2020_9365_MOESM4_ESM.docx]

**Additional file 4: BCTs linked to TDF domains [14]**

| TDF Domain (COM-B) | BCT |
| --- | --- |
| Knowledge (psychological capability) | Links  2.6. Biofeedback  4.1. Instruction on how to perform behaviour  4.2. Information about antecedents  5.1. Information about health consequences  5.2. Salience of consequences  5.3. Information about social and environmental consequences  Inconclusive  2.2. Feedback on behaviour |
| Memory, Attention, Decision Making (psychological capability) | Links  7.1. Prompts/cues  11.3. Conserving mental resources  Inconclusive  1.9. Commitment  7.8. Associative learning  8.4. Habit reversal |
| Behavioural Regulation (psychological capability) | Links  1.2. Problem solving  2.3. Self-monitoring of behaviour  4.2. Information about antecedents  8.2. Behaviour substitution  11.2. Reduce negative emotions  11.3. Conserving mental resources  Inconclusive  1.1 Goal setting (behaviour)  1.4. Action planning  1.6. Discrepancy between current behaviour and goal  1.8. Behavioural contract  2.4. Self-monitoring of outcomes of behaviour  8.3. Habit formation  8.4. Habit reversal  14.2. Punishment |
| Skills (psychological capability) | Links  4.1. Instruction on how to perform behaviour  8.1. Behavioural practice/rehearsal  8.7. Graded tasks  Inconclusive  1.2. Problem solving  6.1. Demonstration of the behaviour  8.6. Generalisation of target behaviour  10.9. Self-reward |
| Social Influences (social opportunity) | Links  3.1. Social support (unspecified)  3.2. Social support (practical)  6.2. Social comparison  6.3. Information about others’ approval  10.4. Social reward  Inconclusive  2.1. Monitoring of behaviour by others without feedback  12.2. Restructuring the social environment |
| Environmental Context and Resources (physical opportunity) | Links  3.2. Social support (practical)  7.1. Prompts/cues  7.5. Remove aversive stimulus  12.1. Restructuring the physical environment  12.2. Restructuring the social environment  12.3. Avoidance/reducing exposure to cues for the behaviour  12.5. Adding objects to the environment  Inconclusive  1.2. Problem solving  11.3. Conserving mental resources |
| Beliefs about consequences (reflective motivation) | Links  5.1. Information about health consequences  5.2. Salience of consequences  5.3. Information about social and environmental consequences  5.5. Anticipated regret  5.6. Information about emotional consequences  9.2. Pros and cons  9.3. Comparative imagining of future outcomes  10.1. Material incentive (behaviour)  10.8. Incentive (outcome)  10.10. Reward (outcome) |
| Beliefs about capabilities (reflective motivation) | Links  1.2. Problem solving  4.1. Instruction on how to perform behaviour  6.1. Demonstration of the behaviour  8.1. Behavioural practice/rehearsal  8.7. Graded tasks  15.1. Verbal persuasion about capability  15.3. Focus on past success  15.4. Self-talk  Inconclusive   - 1. Goal setting (behaviour)   2.6. Biofeedback  10.4. Social reward  11.2. Reduce negative emotions |
| Optimism (reflective motivation) | Inconclusive  1.7. Review outcome goal(s) |
| Social/professional role and identity (reflective motivation) | Inconclusive  3.1. Social support (unspecified)  6.2. Social comparison  9.1. Credible source  13.5. Identity associated with changed behaviour |
| Intentions (reflective motivation) | Links   - 1. Goal setting (behaviour)   5.1. Information about health consequences  10.8. Incentive (outcome)  Inconclusive  1.9. Commitment  6.3. Information about others’ approval  13.4. Valued self-identify |
| Goals (reflective motivation) | Links  1.1. Goal setting (behaviour)  1.3. Goal setting (outcome)  1.5. Review behaviour goal(s)  1.6. Discrepancy between current behaviour and goal  1.7. Review outcome goal(s)  Inconclusive  1.8. Behavioural contract  8.7. Graded tasks  10.2. Material reward (behaviour) |
| Reinforcement (automatic motivation) | Links  10.1. Material incentive (behaviour)  10.2. Material reward (behaviour)  10.3. Non-specific reward  10.4. Social reward  10.6. Non-specific incentive  10.8. Incentive (outcome)  10.10. Reward (outcome)  14.2. Punishment  Inconclusive  2.1. Monitoring of behaviour by others without feedback  2.2. Feedback on behaviour  7.1. Prompts/cues  7.8. Associative learning  10.9. Self-reward |
| Emotions (automatic motivation) | Links  11.2. Reduce negative emotions  Inconclusive  5.5. Anticipated regret  5.6. Information about emotional consequences  12.6. Body changes  13.2. Framing/reframing |
