# Supplementary material for: Influences on NHS Health Check behaviours: a systematic review
Source: BMC Public Health. 2020 Sep 17;20:1359. doi: 10.1186/s12889-020-09365-2 (PMC7495879; doi:10.1186/s12889-020-09365-2)
Supplement: Supplementary file 5 — Additional file 5:. Search terms. [file 12889_2020_9365_MOESM5_ESM.docx]

**Additional file 5: Search terms**

| 1. health check*.tw. |
| --- |
| 2. (diabetes adj3 screen*).tw. |
| 3. (cardiovascular adj3 screen*).tw. |
| 4. CVD screen*.tw. |
| 5. ((preventive or preventative) adj3 screen*).tw. |
| 6. medical check*.tw. |
| 7. NHS Health Check.tw. |
| 8. (diabetes adj2 prevention).tw. |
| 9. (cardiovascular adj2 prevention).tw. |
| 10. CVD prevention.tw. |
| 11. Cardiovascular Diseases/ and Primary Prevention/ |
| 12. 1 or 2 or 3 or 4 or 5 or 6 or 7 or 8 or 9 or 10 or 11 |
| 13. unhealthy behav*.tw. |
| 14. health* behav*.tw. |
| 15. (behav* adj2 change*).tw. |
| 16. (behav* adj2 insight*).tw. |
| 17. (behav* adj2 decision*).tw. |
| 18. self-efficacy.tw. |
| 19. health confidence.tw. |
| 20. (risk adj2 communicat*).tw. |
| 21. referral.tw. |
| 22. patient* adherence.tw. |
| 23. patient* involvement.tw. |
| 24. self-manag*.tw. |
| 25. (health literacy or health literate).tw. |
| 26. patient* experience*.tw. |
| 27. barrier*.tw. |
| 28. facilitator*.tw. |
| 29. (patient* adj3 motivat*).tw. |
| 30. (appointment* adj2 booking*).tw. |
| 31. (blood test* or bloods).tw. |
| 32. (patient adj2 isolation).tw. |
| 33. Attitude to Health/ |
| 34. self efficacy/ |
| 35. communication/ or health behavior/ or health risk behaviors/ or patient compliance/ or patient participation/ |
| 36. "Referral and Consultation"/ |
| 37. Motivation/ |
| 38. "Appointments and Schedules"/ |
| 39. Choice Behavior/ |
| 40. Consumer Behavior/ |
| 41. 13 or 14 or 15 or 16 or 17 or 18 or 19 or 20 or 21 or 22 or 23 or 24 or 25 or 26 or 27 or 28 or 29 or 30 or 31 or 32 or 33 or 34 or 35 or 36 or 37 or 38 or 39 or 40 |
| 42. 12 and 41 |
| 43. (commissioner* or commissioning).tw. |
| 44. manager*.tw. |
| 45. healthcare professional*.tw. |
| 46. (pharmacy or pharmacies or pharmacist*).tw. |
| 47. provider*.tw. |
| 48. (GP or GPs or general pract*).tw. |
| 49. stakeholder*.tw. |
| 50. health personnel/ or pharmacists/ or general practitioners/ |
| 51. Administrative Personnel/ |
| 52. allied health personnel/ or nurses' aides/ or pharmacy technicians/ or physician assistants/ |
| 53. 43 or 44 or 45 or 46 or 47 or 48 or 49 or 50 or 51 or 52 |
| 54. 42 and 53 |
| 55. limit 54 to english language |
| 56. limit 55 to yr="2008 - 2018" |
| 57. remove duplicates from 56 |
