# Supplementary material for: Influences on NHS Health Check behaviours: a systematic review
Source: BMC Public Health. 2020 Sep 17;20:1359. doi: 10.1186/s12889-020-09365-2 (PMC7495879; doi:10.1186/s12889-020-09365-2)
Supplement: Supplementary file 7 — Additional file 7:. APEASE criteria. [file 12889_2020_9365_MOESM7_ESM.docx]

**Additional file 7: APEASE criteria**

| Criterion | Description |
| --- | --- |
| Affordability | Interventions often have an implicit or explicit budget. It does not matter how effective, or even cost-effective it may be if it cannot be afforded. An intervention is affordable if within an acceptable budget it can be delivered to, or accessed by, all those for whom it would be relevant or of benefit. |
| Practicability | An intervention is practicable to the extent that it can be delivered as designed through the means intended to the target population. For example, an intervention may be effective when delivered by highly selected and trained staff and extensive resources but in routine clinical practice this may not be achievable. |
| Effectiveness and cost-effectiveness | Effectiveness refers to the effect size of the intervention in relation to the desired objectives in a real world context. It is distinct from efficacy which refers to the effect size of the intervention when delivered under optimal conditions in comparative evaluations. Cost-effectiveness refers to the ratio of effect (in a way that has to be defined, and taking account of differences in timescale between intervention delivery and intervention effect) to cost. If two interventions are equally effective then clearly the most cost-effective should be chosen. If one is more effective but less cost-effective than another, other issues such as affordability, come to the forefront of the decision making process. |
| Acceptability | Acceptability refers to the extent to which an intervention is judged to be appropriate by relevant stakeholders (public, professional and political). Acceptability may differ for different stakeholders. For example, the general public may favour an intervention that restricts marketing of alcohol or tobacco but politicians considering legislation on this may take a different view. Interventions that appear to limit agency on the part of the target group are often only considered acceptable for more serious problems (19). |
| Side-effects/safety | An intervention may be effective and practicable, but have unwanted side-effects or unintended consequences. These need to be considered when deciding whether or not to proceed. |
| Equity | An important consideration is the extent to which an intervention may reduce or increase the disparities in standard of living, wellbeing or health between different sectors of society. |
